# Pan-3D genome analysis reveals structural and functional differentiation of soybean genomes

**DOI:** 10.1186/s13059-023-02854-8

**Published:** 2023-01-19

**Authors:** Lingbin Ni, Yucheng Liu, Xin Ma, Tengfei Liu, Xiaoyue Yang, Zhao Wang, Qianjin Liang, Shulin Liu, Min Zhang, Zheng Wang, Yanting Shen, Zhixi Tian

**Affiliations:** 1 State Key Laboratory of Plant Cell and Chromosome Engineering, Institute of Genetics and Developmental Biology, Innovative Academy of Seed Design, Chinese Academy of Sciences, Beijing, 100101 China; 2 College of Advanced Agriculture Sciences, University of Chinese Academy of Sciences, Beijing, 100049 China; 3 Beijing Key Laboratory of Vegetable Germplasm Improvement, Beijing Vegetable Research Center, Beijing Academy of Agriculture and Forestry Sciences, Beijing, 100097 China

**Keywords:** Pan-3D genome, Structural variations, Non-LTR retrotransposons, Gypsy elements, Satellite repeats, PAV

## Abstract

**Background:**

High-order chromatin structure plays important roles in gene regulation. However, the diversity of the three-dimensional (3D) genome across plant accessions are seldom reported.

**Results:**

Here, we perform the pan-3D genome analysis using Hi-C sequencing data from 27 soybean accessions and comprehensively investigate the relationships between 3D genomic variations and structural variations (SVs) as well as gene expression. We find that intersection regions between A/B compartments largely contribute to compartment divergence. Topologically associating domain (TAD) boundaries in A compartments exhibit significantly higher density compared to those in B compartments. Pan-3D genome analysis shows that core TAD boundaries have the highest transcription start site (TSS) density and lowest GC content and repeat percentage. Further investigation shows that non-long terminal repeat (non-LTR) retrotransposons play important roles in maintaining TAD boundaries, while Gypsy elements and satellite repeats are associated with private TAD boundaries. Moreover, presence and absence variation (PAV) is found to be the major contributor to 3D genome variations. Nevertheless, approximately 55% of 3D genome variations are not associated with obvious genetic variations, and half of them affect the flanking gene expression. In addition, we find that the 3D genome may also undergo selection during soybean domestication.

**Conclusion:**

Our study sheds light on the role of 3D genomes in plant genetic diversity and provides a valuable resource for studying gene regulation and genome evolution.

**Supplementary Information:**

The online version contains supplementary material available at 10.1186/s13059-023-02854-8.

## Background

Three-dimensional (3D) genome organization can bring distal cis-regulatory elements (CREs) into spatial proximity with their target genes and plays an important role in gene regulation [1–3]. High-throughput studies of chromatin structure have shed light on a hierarchical organization of the 3D genome [4, 5], which can be arranged into chromosome territories, compartments, topologically associating domains (TADs), and chromatin loops [6–8]. The compartments represent dynamic but nonrandom hierarchical structures characterized by stretches of megabase-long transcriptionally active A compartments that are interspersed with transcriptionally inactive B compartments [9–12]. TADs are defined as contiguous genomic segments in which DNA sequences exhibit significantly higher interaction frequencies than those outside of TADs [7, 13–15]. TADs are separated by TAD boundaries, and these boundaries can restrict CRE interactions with promoters within TADs [16, 17].

Comparative analyses have suggested that chromatin structure changes may serve as an important driving force during cell differentiation, embryo development, and genome evolution [10, 12, 18–23]. For example, a comparison among humans, macaques, and mice identified a number of human-specific sequences around human-specific TAD boundaries, which generated many new transcription factor binding sites and human-specific chromatin structures [18]. Furthermore, it was found that genomic structural variations (SVs) may lead to the rearrangement of 3D genome, which may result in subsequent expression changes in some genes [24–28]. For example, in humans, a large deletion including CCCTC-binding factor (CTCF)-associated boundary elements at the *EPHA4* locus results in the ectopic interaction of an enhancer cluster and genes that are normally separated, causing misexpression and congenital limb malformation [29]; in maize, the transposable element (Hopscotch) that inserted in the regulatory region of *teosinte branched1* (*tb1*), a maize domestication gene, can act as an enhancer to increase the expression of *tb1*, which partially explains the increased apical dominance in maize compared to its progenitor, teosinte [30].

Genetic variation is the engine that propels plant breeding to meet future challenges [31–33]. Although previous genomic studies in plant genetic resources have provided critical biological insights [31, 34–36], most aimed at genomic sequence information and our understanding of the 3D genomic diversity among plant accessions remains limited. In addition, due to the lack of high-quality SVs data, the impact of SVs on the 3D genome has not been fully evaluated so far, particularly in plants. In this study, we took advantage of the 27 soybean accessions for which we previously conducted de novo genome assembly [37–39]. Given these 3D genomic information based on custom genomes, we performed pan-analyses and constructed the pan-3D genome of A/B compartments and TAD boundaries. The pan-3D genome showed that compartment reorganizations were associated with I regions. Moreover, it was found that non-long terminal repeat (non-LTR) retrotransposons maintained TAD boundaries, while Gypsy elements and satellite repeats established private TAD boundaries. Further assessment of the effects and contributions of SVs to 3D genome variation showed that presence and absence variations played important role in 3D genome variations. Expression analyses demonstrated the functional effect of pan-3D genome. Our study provides a deeper insight into genome evolution and gene regulation in plant accessions.

## Results

### Hi-C sequencing of 27 soybean accessions

To investigate the 3D genome organization in soybean, we performed in situ Hi-C sequencing of the 27 accessions which included 3 wild soybeans, 9 landraces, and 15 improved cultivars [37–39]. The in situ Hi-C experiment for each accession was designed with two biological replicates, and each replicate produced an average of ~470 million raw read pairs and ~100 million unique long-range (more than 20 kb) cis contacts (Additional file 1: Fig. S1a, b; Additional file 2: Table S1). The two biological replicates of each accession were highly reproducible (Additional file 1: Fig. S1c). Contact maps showed relatively independent interactions within the pericentromeric heterochromatin and euchromatin arms and intense interactions along anti-diagonal lines (Additional file 1: Fig. S1d, e), consistent with previous results [40]. Complementing these Hi-C data, we profiled the transcriptome through RNA sequencing (RNA-seq) in these accessions.

To determine the power of the Hi-C data to reflect the 3D genome organization with a consideration of the possible influence of read depth across individual accessions, we inspected the contact matrix resolution using the valid pairs in bins [8] as well as 3D chromatin structure (Additional file 1: Fig. S1f–i). We determined that saturation of the 3D chromatin structure occurred in a 25-kb contact map (Additional file 1: Fig. S1f, i). Therefore, the robust contact matrix resolution of 25 kb was adopted in this study.

### Identification of A/B compartments in individual accessions

**Fig. 1:**
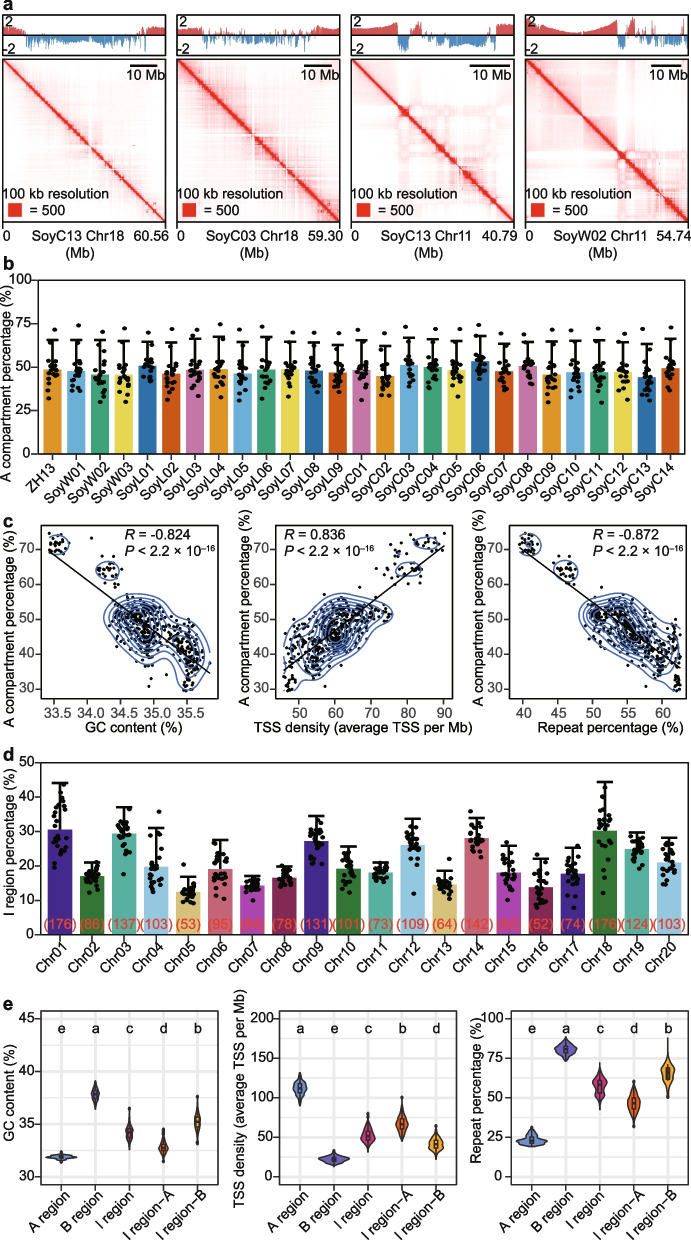
Characteristics of A/B compartments and I regions in 27 soybean accessions. **a** Examples of Hi-C contact maps and E1 value tracks of chromosomes in soybean accessions. The red bars of E1 value tracks represent the A compartments, and the blue bars of E1 value tracks represent the B compartments. **b** A compartment percentages across chromosomes in 27 soybean accessions. Each dot represents one chromosome. Data are mean ± s.d. **c** Correlation analysis of A compartment percentages across chromosomes and GC contents, TSS densities and repeat percentages. **d** I region percentages across chromosomes in 27 soybean accessions. Number in parentheses indicates the average number of I regions. Each dot represents one accession. Data are mean ± s.d. **e** Violin plot of GC contents, TSS densities, and repeat percentages across chromosomes in A regions, B regions, I regions, I regions-A (A compartments in I regions) and I regions-B (B compartments in I regions) in 27 soybean accessions. Boxes represent the 25th, 50th, and 75th percentiles, and whiskers represent 1.5× the interquartile range. Multiple comparisons were performed with one-sided Wilcoxon rank-sum test with Benjamini–Hochberg multiple testing correction

Using the in situ Hi-C sequencing data, we checked the organization of A/B compartments (Additional file 2: Table S2) and found certain differentiation of A/B compartments within chromosomes. For example, we observed differences around pericentromeric heterochromatin of chromosome 18 in SoyC13 and SoyC03, and variations in the euchromatin arms of chromosome 11 in SoyW02 and SoyC13 (Fig. 1a). Despite these differences, the average A compartment percentages were relatively consistent across individual accessions (Fig. 1b). Collectively, these results reveal that the overall percentages of compartments are conserved, but the reorganization of A/B compartments within chromosomes occurs frequently in soybean accessions.

We subsequently checked the relationship between A/B compartments and genomic features in soybean. As expected, strong positive correlations were observed between the A compartment percentage and transcription start site (TSS) density, while strong negative correlations of the A compartment percentage with the GC content and repeat percentage were observed (Fig. 1c). Furthermore, comparative analysis showed that B compartments exhibited significantly higher GC contents, higher repeat percentages, and lower TSS densities than the A compartments (Additional file 1: Fig. S2a–c). Nevertheless, we surprisingly found that the repeat percentage of the A compartment on several chromosomes was significantly higher than the average level, such as chromosome 1 of SoyC06 (Additional file 1: Fig. S2d). Tracing these A compartments showed that the highly repetitive regions mainly came from the regions of intersection between A compartments and B compartments (Additional file 1: Fig. S2e, f), which were referred to as I regions in this study. We found substantial differences in the percentages of I regions either across accessions or across chromosomes, among which chromosome 1 showed a higher percentage of I regions (Fig. 1d). A comparison among the I regions, A regions (A compartments removing I regions) and B regions (B compartments removing I regions) of chromosome 1 in SoyC06 showed that the repeat percentage of I regions presented an intermediate value (Additional file 1: Fig. S2g). Additionally, the A compartments and B compartments from I regions also presented intermediate status (Additional file 1: Fig. S2h). Further investigation using the data from 27 accessions revealed that the GC contents, TSS densities, and repeat percentages of I regions (including the A compartments or B compartments from I regions) exhibited an intermediate status between the A regions and the B regions (Fig. 1e). Overall, these data illustrate that I regions exhibit intermediate genomic features.

### Pan-analyses of A/B compartments across the 27 soybean accessions

**Fig. 2:**
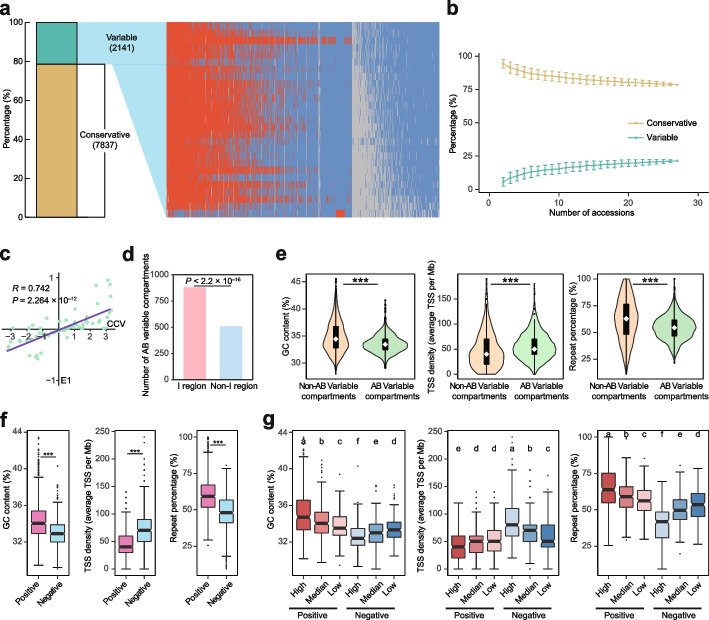
Pan-3D genome of A/B compartments. **a** Bar plot indicates the percentages of conservative and variable compartments. Heatmap showing the distribution of variable compartments across 27 soybean accessions. The colors reflect the compartment types of the compartment bins, where red bins represent A compartments, blue bins represent B compartments and gray bins represent NAs. Each row of the heatmap represents one soybean accession, and each column of the heatmap represents one compartment bin. The accessions from top to bottom are SoyC02, SoyC07, SoyC06, SoyL08, SoyL09, SoyC13, SoyC12, SoyL05, SoyL04, SoyL06, ZH13, SoyL03, SoyC05, SoyC14, SoyC04, SoyL07, SoyW03, SoyC11, SoyL02, SoyC10, SoyC08, SoyC09, SoyL01, SoyC01, SoyC03, SoyW01, and SoyW02. **b** Simulation of the conservative and variable compartments based on 100 randomizations of soybean accessions. Data are mean ± s.d. **c** Correlation analysis of CCVs and average E1 values. **d** Distribution of AB variable compartments in I regions and non-I regions. *P* value was calculated by one-sided Fisher’s exact test. **e** Violin plot of GC contents, TSS densities and repeat percentages of AB variable compartments and non-AB variable compartments in I regions. ***P < 0.001 for comparisons between AB variable compartments and non-AB variable compartments (two-sided Wilcoxon rank-sum test). The white boxes in the violin plot represent medians. **f** Box plot of GC contents, TSS densities, and repeat percentages in AB variable compartments with negative and positive CCVs. *P* values were calculated by one-sided Wilcoxon rank-sum test. **g** Box plot of GC contents, TSS densities, and repeat percentages in AB variable compartments with high (the high 30%), median (the median 40%), and low (the low 30%) absolute values of positive and negative CCVs. Multiple comparisons were performed by one-sided Wilcoxon rank-sum test with Benjamini–Hochberg multiple testing correction

We performed pan-analyses of A/B compartments through the investigation of their conservation and variation across the 27 soybean accessions. It showed that 78.5% of the compartment bins in the genome exhibited conservation (Fig. 2a; Additional file 2: Table S3). Modeling the pan-3D genome size by iteratively randomly sampling accessions suggested a closed pan-3D genome with finite numbers of both conservative and variable compartments (Fig. 2b), which showed a similar pattern to our previous pan-genome analyses [39]. The A compartments showed a higher ratio of conservative compartments than B compartments (Additional file 1: Fig. S3a). We then inspected the variable compartments by dividing them into three types: compartments with only A compartments, compartments with only B compartments, and compartments with both A and B compartments (AB variable compartments). A large proportion (64.83%) of the compartment variation occurred in AB variable compartments, indicating extensive compartment switching across soybean accessions (Additional file 1: Fig. S3b).

Next, we examined the distribution and variation of AB variable compartments. To observe the variation tendency, we calculated a “coefficient of compartment variation” (CCV), which was defined as the natural logarithm of the number of B compartments divided by the number of A compartments in AB variable compartment bins. We further plotted AB variable compartments with CCVs throughout the genome and observed that AB variable compartments were mainly distributed in regions of A/B compartment intersection, and the CCVs appeared to correlate with E1 values—the first eigenvalues in the eigenvector decomposition of compartments (Additional file 1: Fig. S3c). Further analyses showed that there was a strong correlation between E1 and CCVs (Fig. 2c), and AB variable compartments were significantly enriched in I regions (Fig. 2d). Previous reports showed that E1 was highly correlated with GC content [41]; hence, we investigated the relationship between CCVs and genomic features. Accordingly, CCVs were positively correlated with GC content and repeat percentage and negatively correlated with TSS density (Additional file 1: Fig. S3d), which was in accord with the observation that regions with intermediate genomic features were more likely to undergo A/B compartment switching. We then attempted to decode the differences between AB variable compartments and non-AB variable compartments in I regions and observed that AB variable compartments had higher TSS densities and lower GC contents and repeat percentages (Fig. 2e).

We further analyzed the genomic features of AB variable compartments with different CCVs. First, we compared the AB variable compartments with positive and negative CCVs and found that the positive CCV group showed a higher TSS density and lower GC content and repeat percentage than the negative CCV group (Fig. 2f). Furthermore, according to the absolute value of CCVs, we divided each AB variable compartments with positive and negative CCVs into three subgroups: low, median, and high. We found that the groups with lower CCV absolute values were more likely to show an intermediate GC content, TSS density, and repeat percentage (Fig. 2g). Therefore, these results indicate that regions with intermediate genomic features show the highest variation of compartments among the 27 accessions.

### TAD identification and characterization in individual soybean accessions

To identify TADs in soybean, the reported “insulation score” method [16] was adopted in this study (Additional file 2: Table S4). In parallel, contact domains and directionality indexes were also calculated [7, 8]. Although boundaries exhibited some variations across the 27 accessions, they had comparable normalized numbers (Additional file 1: Fig. S4a). The identification results showed that the median size of TADs in soybean was approximately 475 kb (Additional file 1: Fig. S4b).

**Fig. 3:**
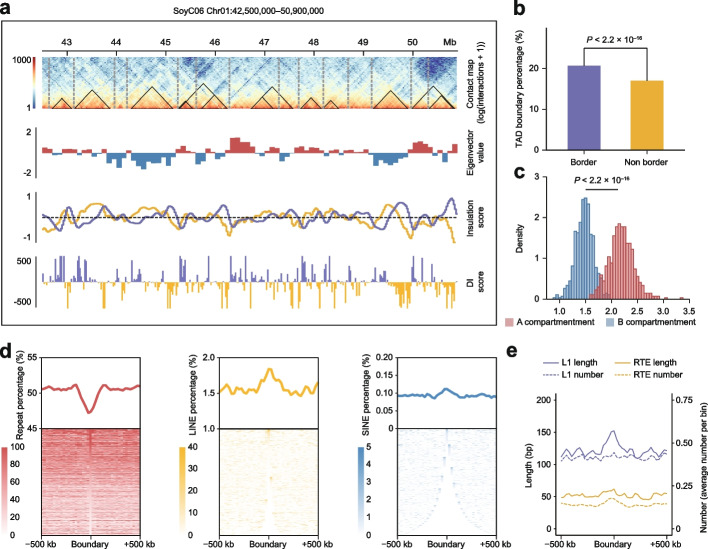
TADs in 27 soybean accessions. **a** Hi-C contact map of the 42.5–50.9 Mb region of chromosome 1 in SoyC06, along with eigenvectors, insulation scores, and DI scores. Solid black triangles represent the contact domains identified by Arrowhead, and dashed gray lines represent TAD boundaries. The insulation profile is depicted in yellow and the insulation “delta” vector is depicted in purple in the insulation score panel. **b** TAD boundary percentage with compartment borders and non-compartment borders. The *P* value was calculated by two-sided Fisher’s exact test. **c** Bar plot of the TAD boundary density in A compartments and B compartments in 27 soybean accessions. *P* value was calculated by Kolmogorov–Smirnov test. **d** Enrichment of repeat elements, LINE elements, and SINE elements around TAD boundaries in 27 soybean accessions. **e** Enrichment of the lengths and numbers of L1 and RTE fragments around TAD boundaries in 27 soybean accessions

We first investigated the distribution of boundaries and A/B compartments and observed that numbers of boundaries overlapped with compartment borders (Fig. 3a). For validation, we confirmed that boundaries were significantly enriched in compartment borders (Fig. 3b), indicating that compartmentalization may be an important driver of TAD formation in soybeans. In addition, we found that the density of boundaries in A compartments was significantly higher than that in B compartments (Fig. 3c). Taken together, these results highlight the important role of compartmentalization in TAD formation in soybeans.

Repeats, including transposable elements (TEs) and tandem repeats (TRs), have been universally found in eukaryotic species and display extreme diversity [42, 43]. Based on their unique structures, TEs can be classified into different classes and superfamilies [44]. In humans, non-LTR TEs, including long interspersed nuclear elements (LINEs) and short interspersed nuclear elements (SINEs), account for the majority of TEs [45–47]. It was found that LINEs and SINEs exhibited enrichment around boundaries in humans and mice [7, 18, 23]. The relationship between repeats and TADs in plants is largely unknown.

We analyzed the enrichment of repeats around TAD boundaries. Meta-analysis revealed that repeats were depleted around boundaries (Fig. 3d). In contrast to humans, LTRs are the dominant TEs in plants [48, 49]. In soybean, non-LTR TEs accounted for less than 5% of the TEs, while LTRs accounted for ~80% (Additional file 1: Fig. S4c). Subsequently, we investigated the enrichment of different repeat types. Interestingly, although most repeats were found to be depleted around boundaries, LINEs and SINEs were found to be enriched around boundaries particularly (Fig. 3d; Additional file 1: Fig. S4d). These results were further confirmed through the analyses of each of the 27 soybean accessions (Additional file 1: Fig. S4e, f). It has been found that most identified LINEs in plants come from the L1 and RTE superfamilies [50, 51]. Enrichment analysis of superfamilies showed that the length of the L1 fragment was longer around boundaries (Fig. 3e).

### Pan-analyses of TADs across the 27 soybean accessions

**Fig. 4:**
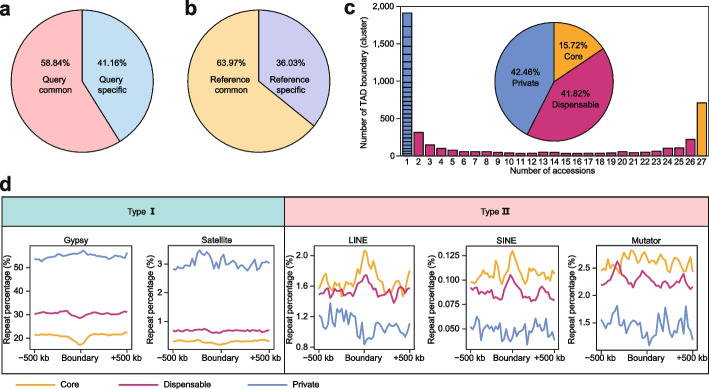
Pan-3D genome of TAD boundaries. **a** Average percentages of common and specific TAD boundaries in query accessions in the comparative 3D genome analysis. **b** Average percentages of common and specific TAD boundaries in ZH13 accession in the comparative 3D genome analysis. **c** Pan-3D genome of TAD boundaries. Pie chart showing the proportion of core, dispensable, and private TAD boundaries (clusters) in the pan-3D genome analysis. When the accession number is 1, each box in the bar represents one accession. **d** Enrichment of repeat elements around core, dispensable, and private TAD boundaries of different types in 27 soybean accessions

To investigate the TAD dynamics across soybean accessions, we performed comparative 3D genome analysis using ZH13 as the reference and the other 26 accessions as queries. The results revealed that on average 58.84% of the boundaries in query accessions were common relative to those in reference accession, while 63.97% of the boundaries in reference accession were common relative to those in query accessions (Fig. 4a, b).

We performed the pan-3D genome analysis of boundaries by adopting a method combining the alignment and clustering of boundaries across 27 accessions (Additional file 1: Fig. S5a). We found that nearly 20% (708/4,505) of the boundary clusters were categorized as core types, 41.82% (1884/4505) were categorized as dispensable types, and 42.46% (1913/4505) were categorized as private types (Fig. 4c; Additional file 2: Table S5). GO analysis showed that the core types were mainly involved in basic metabolism and transcription processes, while the dispensable types were involved in various noncoding RNAs and posttranscriptional RNA processing (Additional file 1: Fig. S5b, c). No significant enrichment was found for the private types, indicating that private boundaries may participate in specific biological functions.

Genomic feature analyses demonstrated that the common/core boundaries had the highest TSS density and lowest GC content and repeat percentage (Additional file 1: Fig. S5d, e). We then investigated the enrichment of different repeat types around boundaries of the pan-3D genome. Surprisingly, two opposite patterns were observed (Fig. 4d). The first pattern showed a higher proportion of repeat elements in the specific boundaries than in the core and dispensable boundaries, such as Gypsy elements and satellite repeats (Fig. 4d), indicating that these two types of elements may play an important roles in specific boundary formation. In contrast, the second pattern showed a higher proportion of repeats in the core boundaries, including LINEs, SINEs, and all DNA transposons except Helitron elements (Fig. 4d; Additional file 1: Fig. S5f). Additionally, the distributions of several repeat types, such as Copia elements and Helitron elements, seemed to be unclear (Additional file 1: Fig. S5g). Moreover, we found that LINEs and SINEs were enriched around the boundaries of core and dispensable types but showed a depleted pattern around specific boundaries (Fig. 4d).

### Genomic SVs in the 3D genome

SVs, including presence and absence variations (PAVs), copy number variations (CNVs), inversions (INVs), and translocations (TRANSs), are universally present across the accessions of a given species [52–55]. It has been reported that SVs can influence the 3D genome organization; however, apart from studies of specific loci, little is known about these effects at the genome-wide level [29, 56–58]. Coupled with high-quality SVs data from de novo assembled genomes, our data enabled us to conduct a comprehensive analysis between 3D genome variations and SVs.

**Fig. 5:**
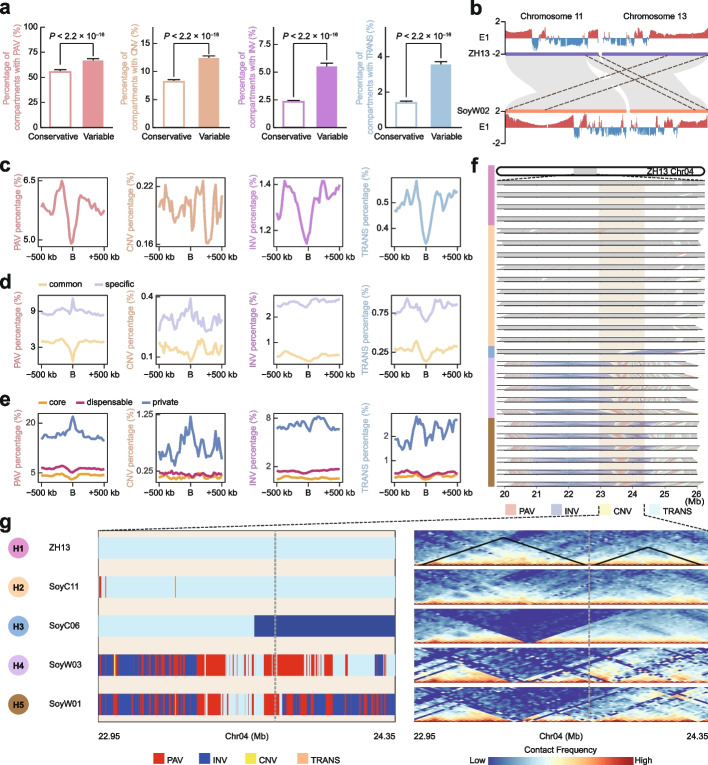
Structural variations in the 3D genome. **a** Percentages of conservative and variable compartments with PAV, CNV, INV, and TRANS. *P* values were calculated by one-sided Fisher’s exact test. **b** Example of A/B compartments and structural variations. The pink colored lines represent large translocations between chromosome 11 and chromosome 13. **c** Percentages of structural variations around TAD boundaries. **d** Percentages of structural variations around common and specific TAD boundaries. **e** Percentages of structural variations around core, dispensable, and private TAD boundaries. **f** Snapshot of the structural variation catalog between the ZH13 genome and 26 query accessions. The brown box represents the 22.95–24.35 Mb region of Chr04 in ZH13. The color bars on the left represent five haplotypes in 27 accessions. The 26 collinear panels represent the collinearity and structural variations around the region, where the bottom line represents the ZH13 accession, and the top line represents the query accession in each panel. These query accessions from top to bottom are SoyL01, SoyL04, SoyC04, SoyC05, SoyC11, SoyC03, SoyC08, SoyL07, SoyC09, SoyC13, SoyC14, SoyL06, SoyC02, SoyC07, SoyC06, SoyW03, SoyL02, SoyL08, SoyC10, SoyC12, SoyW01, SoyW02, SoyL03, SoyL05, SoyL09, and SoyC01. **g** Examples of five haplotypes and Hi-C contact maps. The left panel represents the structural variations of the five haplotypes relative to ZH13, and the right panel represents the Hi-C contact maps of the five haplotypes in ZH13

We first investigated the distributions of SVs in A/B compartments. Comparative analyses revealed that the four types of SVs showed significantly higher ratios in the variable compartments than in the conservative compartments (Fig. 5a). Further investigation showed that PAVs were more likely to occur in A compartments than the other SVs (Additional file 1: Fig. S6a), whereas no obvious preference in the distribution of SVs was found in I regions (Additional file 1: Fig. S6b). In addition, although some SVs may not change the properties of A/B compartments, they affected the observed E1 values, taking the two large translocations on chromosome 11 and chromosome 13 in SoyW02 as examples (Fig. 5b). For the first translocation pair (beginning ~20 Mb of SoyW02 chromosome 11 vs. end ~20 Mb of ZH13 chromosome 13), the average E1 value from SoyW02 for this region was approximately 25% higher than that of ZH13 (0.65 ± 0.40 vs. 0.51 ± 0.18; mean ± s.d; *P* = 1.295×10^−5^, paired *t*-test). For the second translocation pair (end ~5 Mb of SoyW02 chromosome 13 vs. end ~5Mb of ZH13 chromosome 11), although the E1 values from these two regions did not show significant difference (0.81 ± 0.26 vs. 0.84 ± 0.19; mean ± s.d; *P* = 0.068, paired *t*-test), but the E1 values from the last three windows of SoyW02 were approximately doubled compared to that of ZH13 (Fig. 5b).

Next, we investigated the distribution of SVs around TAD boundaries. Four types of SVs were clearly depleted around boundaries, consistent with previous studies showing that TAD boundaries were relatively conserved [7] (Fig. 5c). Similar to the distributions observed in A/B compartments, the SVs showed a significantly higher proportion in specific boundaries or private boundaries (Fig. 5d, e). Additionally, despite the depletion of SVs around boundaries, obvious peaks of PAVs and CNVs were observed around specific boundaries or private boundaries, indicating that these unbalanced rearrangements may participate in boundary formation in soybean.

As an example, the boundary between two TADs in the ~1.4 Mb region on chromosome 4 was significantly affected by SVs. We observed five haplotypes of SVs in this region, where haplotype 1 showed collinearity and haplotype 2 contained intra-TAD SVs, haplotype 3 contained a large INV on the boundary, haplotype 4 contained a PAV on the boundary and an intra-TAD INV, and haplotype 5 contained a PAV on the boundary and two intra-TAD INVs (Fig. 5f). Haplotype 3, 4, and 5 consistently caused the loss of the boundary in 12 accessions. These results were further supported by Hi-C contact maps (Fig. 5g). These data collectively support that SVs reshape the 3D genome across soybean accessions.

### Effect and contribution of SVs to 3D genome variations

Despite the fateful effect of SVs on TAD boundary in the provided example, these effects of SVs are largely unknown throughout the genome. Additionally, such contributions of SVs to 3D genome variation have yet to be elucidated. To this end, we investigated the effects and contributions of SVs in a genome-wide manner.

**Fig. 6:**
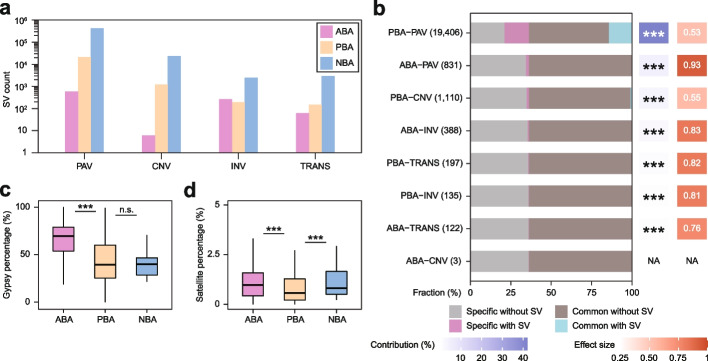
Contributions and effects of structural variations. **a** Numbers of structural variations of the ABA, PBA, and NBA types. ABA, absolute boundary-affecting. PBA, partial boundary-affecting. NBA, non-boundary-affecting. **b** Bar plot showing the percentages of TAD boundaries of different types. The box behind each bar represents the effect size and contribution. The contribution of each SV is defined as the proportion of specific TAD boundaries that can be explained by each type. The effect size is defined as the proportion of specific TAD boundaries with the SV relative to all the TAD boundaries with the SV. The number of analyzed SVs of each type is shown in parentheses. *P* values were calculated by one-sided Fisher’s exact test. ***P < 0.001. NA, not applicable. **c** Percentages of Gypsy elements of ABA, PBA, and NBA SVs. *P* values were calculated by one-sided Wilcoxon rank-sum test. ***P < 0.001. n.s., not significant. **d** Percentages of satellite repeats of ABA, PBA, and NBA SVs. *P* values were calculated by one-sided Wilcoxon rank-sum test. ***P < 0.001

We first divided SVs into three types: absolute boundary-affecting (ABA) SVs were defined as SVs spanning the whole length of one boundary, partial boundary-affecting (PBA) SVs were defined as SVs only spanning part of the length of one boundary, and non-boundary-affecting (NBA) SVs were defined as SVs within TADs. Most SVs belong to the NBA type, consistent with the finding that SVs are rare around boundaries (Fig. 6a).

We next investigated the contributions of these different types of SVs to 3D genome variation (Fig. 6b). Each type of SVs contributed to boundary variations significantly. Nevertheless, less than half of the boundary variations could be independently explained by SVs, among which PBA-PAV showed the largest (42.05%) contribution. Additionally, ABA-PAV contributed 4.97% of the boundary variations, which was second only to the contribution of PBA-PAV. These results indicated that PAVs, not CNVs, INVs, or TRANSs, played the most important roles in boundary variations. Next, we checked the effect of SVs on boundary variations. As expected, ABA-PAV showed the largest effect on boundary variations. Interestingly, for most balanced rearrangements, such as INVs or TRANSs, we did not observe extensive conservation of boundaries with either ABA types or PBA types, implying that boundaries were not solely dependent on sequence conservation during genomic rearrangement across soybean accessions. Together, these results reveal that at the global level, there are significant correlations between SVs and 3D genome variations but relatively finite changes of 3D genomes, indicating that SVs play a contributory but not deterministic role in 3D genome variations.

TE insertion is an important contributor driving SV formation [59, 60]. Hence, we investigated the effects of TEs in driving SVs causing 3D genome variation. For most TEs, we did observe a lower percentage in ABA or PBA SVs than in NBA SVs (Additional file 1: Fig. S6c). Similar patterns were also observed for most TRs (Additional file 1: Fig. S6c). Only Gypsy elements and satellite repeats showed extremely significant enrichment in ABA SVs relative to PBA SVs, consistent with the results showing that these two repeats were enriched in private boundaries (Fig. 6c, d).

### 3D genome variation and gene expression

To address the effect of 3D genome variation on gene expression, we first investigated the relationship between gene expression and A/B compartments. Consistent with previous studies, genes in A compartments showed higher expression than those in B compartments (Additional file 1: Fig. S7a). In addition, significant expression differences were found between A regions, B regions, I regions, A compartments in I regions, and B compartments in I regions, revealing synergistic expression in I regions (Additional file 1: Fig. S7b). At the pan-3D genome level, genes in conservative compartments showed higher expression than those in variable compartments (Additional file 1: Fig. S7c). These expression differences were also observed in compartments with positive and negative CCVs (Additional file 1: Fig. S7d) and subtypes of these compartments (Additional file 1: Fig. S7e). These results collectively show that gene expression is associated with compartment types, implying the inherent connections among compartments, genomic features, and gene expression.

Next, we examined the relationship between gene expression and TADs. Gene expression in common or core boundaries was higher than that in specific or private boundaries (Additional file 1: Fig. S8a, b). Additionally, we observed the distribution of differentially expressed genes (DEGs) in the 3D genome. Enrichment of DEGs was observed around specific or private boundaries, while the density of DEGs in common or core boundaries was higher than that in specific or private boundaries (Additional file 1: Fig. S8c, d). These data are in accord with the classification of TAD boundaries, indicating the functional effect of boundaries in comparative 3D genomes and pan-3D genomes.

To exclude the potential influence of genomic variations within gene bodies, we extracted “nonVariation-genes” whose genic regions did not overlap with any genomic variations. Nearly half (45.2%) of these nonVariation-DEGs were associated with boundary variations (Additional file 1: Fig. S8e; Additional file 2: Table S6), implying the distinct 3D genome effect of these nonVariation-DEGs in soybean. We further detected specific cases by correlating the three-dimensional genome variation to the gene expression variation. For example, *SoyZH13_09G153300* did not show obvious genetic variations in SoyC10 and SoyC11, but SoyC10 and SoyC11 showed significant lower gene expression than the other accessions (Additional file 1: Fig. S9a). Pan-3D genome showed that SoyC10 and SoyC11 lost one TAD boundary, which was ~60 kb away from this gene. As a validation, the Hi-C contact map showed that these losses led to a significant reduction in interaction, indicating it might cause the expression change in these two accessions. Another example was *SoyZH13_19G056700*, of which loss of one TAD boundary may also result in a lower expression of this gene (Additional file 1: Fig. S9b).

### 3D genome dynamics during domestication and improvement

It has been reported that cultivated soybean was domesticated from wild soybean (*G. soja* Sieb. & Zucc.) in China 5000 years ago. Apart from studies of genetic variations in the genome, little is known about the variability in higher-order chromatin structure during domestication and improvement in soybeans. To investigate this, we performed comparative analysis of the 3D genome variations in the wild soybeans (*G. soja*), landraces, and improved cultivars.

Using the pan-3D genome data, we observed a core set of over 1800 TAD boundary clusters shared by all three soybean groups. In addition, 457, 521, and 1091 specific TAD boundary clusters were identified for individual *G. soja*, landrace, and improved cultivar groups (Additional file 1: Fig. S10a). Combining with pairwise population differentiation level (*F*_*ST*_) across different soybean groups, we found the selection of TAD boundaries was higher than that of non-boundaries during the soybean domestication, whereas no significant difference during improvement (Additional file 1: Fig. S10b). Further investigation suggested that the genes around selected TAD boundaries had higher expression levels than those of unselected TAD boundaries (Additional file 1: Fig. S10c).

We found the expression variations of some domesticated genes were closely related to the 3D genomic variations. For example, *SoyZH13_14G139200* was located in a domesticated selection sweep region (Additional file 1: Fig. S10e). Structural variations led to the loss of the TAD boundary in two wild soybeans SoyW02, SoyW03, and one landrace SoyL02 (Additional file 1: Fig. S10d and e). As a result, we observed that the *SoyZH13_14G139200* showed significant higher expression level in these 3 accessions than the others. These data suggested that 3D genome structure variation may also play an important role in soybean domestication and improvement.

## Discussion

Detailed characterization of genetic diversity is crucial in crop improvement. In this study, we integrated 3D genomes across 27 soybean accessions and constructed the first pan-3D genome in soybeans. We confirmed that A/B compartment switching is highly dependent on genomic features among 27 accessions. In this regard, these results are similar to previous findings in human colorectal cancer showing that a compartment at the interface between the canonical A and B compartments is reorganized in tumors [61]. Coupled with SV data derived from de novo genome assembly, we assessed their effects on and contributions to 3D genomes in a genome-wide manner. These data represent one of the first studies of 3D genomic diversity investigations across multiple accessions in a single plant species, as well as analysis between 3D genome variations and genomic SVs.

Waves of expansion and contraction in numbers of TEs can result in dramatic differentiations in the overall architecture of the genomes of even closely related plant species, which represent a rich and constantly changing pool of genetic and epigenetic variation on which selection can operate [49]. In plants, with several suggestive examples present the regulatory effect of TE insertions [62, 63], little is known about the role of TEs in higher-order chromatin structure. Although non-LTR retrotransposons only account for a small proportion of TEs in plants and are a dominant TE component in many mammalian genomes [44], and CTCF is absent in plants [64], we surprisingly observed the enrichment of non-LTR retrotransposons around TAD boundaries in soybean. Furthermore, benefiting from the pan-3D genome, we provided multiple lines of evidence showing that Gypsy LTRs and satellite repeats established private TAD boundaries in soybeans. Considering the role of Gypsy insulator in establishing higher-order chromatin domains in *Drosophila* [65], and human endogenous retrovirus subfamily H (HERV-H) may create new TAD boundaries in primate [19], these results highlight the unique role of these LTRs in establishing novel TAD boundaries. Moreover, our data raise the possibility that there may be a conserved mechanism of TAD boundary maintenance and establishment in plants and mammals.

Due to technical limitations, the generalizability of the results is limited by the examination of a single tissue type in 27 accessions, and a comprehensive 3D genome map of multiple tissues and developmental stages may provide more detailed information. Several works suggested that the application of 3D genome modifications in crop breeding may be highly valuable [66–68].

## Conclusion

Our study provides the pan-3D genome landscape of soybean and associates 3D genome variation with genomic SVs and gene expression changes among soybean accessions. These data shed light on the evolutionary dynamics of 3D genomes among plant accessions and may provide another solution for plant breeding by modifying the 3D genome structure.

## Methods

### Plant materials and growth conditions

The 27 soybean accessions used in this study include 3 wild soybeans, 9 landraces, and 15 improved cultivars, which were the same accessions used in our previously reported pan-genome analyses of wild and cultivated soybeans [39]. Because the seeds of wild soybeans show the property of strong dormancy, their seed coat needed to be scarified to break dormancy before planting. The soybean seeds were sown and germinated in standard soil in the greenhouse, and the plants were grown under long-day conditions (16-h light/8-h dark). Daytime and nighttime temperatures were 26–28°C and 18–20°C, respectively, with a relative humidity of 40–60%.

### Tissue collection

Fully expanded young leaves from 2-week-old seedlings were collected for Hi-C and RNA-seq experiments. For the Hi-C experiment, the collected tissues were washed three times with distilled water and were immediately used for subsequent library preparation. For the RNA-seq experiment, after collection, the leaves were immediately shock-frozen in liquid nitrogen and stored at −80°C until subsequent processing.

### In situ Hi-C

In situ Hi-C was performed following previous description [8] with minor modifications. Fresh leaves were cut into 2- to 3-mm strips and cross-linked with 1% formaldehyde at room temperature. The cross-linked tissues were treated with vacuum three times under a vacuum pressure of −60 to −80 kPa, maintained for 10 min each time, after which atmospheric pressure was restored. Subsequently, a final concentration of 0.125 M glycine was added for 5 min at room temperature to terminate fixation. The fixed tissues were homogenized with liquid nitrogen and resuspended in nuclei isolation buffer. The isolated nuclei were lysed with 0.1% SDS at 65°C for 10 min and digested with DpnII at 37°C overnight. The restriction fragments were blunt-ended and biotinylated with biotin-14-dCTP, diluted, and ligated with T4 ligase at 16°C for 4 h. After the reversal of crosslinks at 65°C overnight, the ligated DNA was purified and sheared to lengths of 300–500 bp with Covaris M220, pulled down with streptavidin beads, and prepared for Illumina sequencing according to the standard Illumina library construction protocol. Libraries were quantified and sequenced using Illumina NovaSeq 6000 or HiSeq X Ten sequencing platform.

### RNA-seq

Total RNA was extracted using the RNAprep Pure Plant Kit (Tiangen) according to the manufacturer’s instructions and quantified with NanoDrop spectrophotometer. RNA-seq libraries were constructed using the Illumina TruSeq Stranded mRNA Library Preparation Kit using 1–2 μg of total RNA and were then subjected to sequencing on the Illumina NovaSeq 6000 or HiSeq X Ten sequencing platform.

### Hi-C data processing

The HiC-Pro suite (v.2.9.0) was used for valid mapping of the Hi-C reads [69]. Clean Hi-C data from individual accessions were mapped to their own assembled genomes (termed Self mapping in this study) and a common reference genome, ZH13 genome version 2 (ZH13 v2) (termed Reference mapping in this study), using Bowtie2 (v2.3.3) [70]. During mapping, the reads were first aligned using an end-to-end aligner, and the reads spanning ligation junctions were trimmed at their 3′ end and realigned to the genome. The aligned reads of both fragment mates were then paired in a single paired-end BAM file. Dangling-end reads, same-fragment reads, self-circled reads, self-ligation reads, and other invalid Hi-C reads were discarded from subsequent analyses.

After removing duplications, valid pairs were summed across two biological replicates and were used to generate raw Hi-C matrix at 5 kb, 10 kb, 25 kb, 40 kb, 50 kb, 100 kb, and 1 Mb resolutions. These matrices were normalized by the Knight-Ruiz (KR) method or the iterative correction and eigenvector decomposition (ICE) method. To investigate the contact domains, the valid pairs were converted into .hic format files with the juicer (v1.9.8) tool [71] pre command; to calculate compartment signals, the valid pairs were converted into .cool format files with hicConvertFormat command in HiCExplorer suite (v3.3.1) [72]; and to visualize the Hi-C contact map, the valid pairs were converted into .h5 format.

### Reproducibility score

The reproducibility score was calculated using GenomeDISCO software [73]. Under this method, random walks on the contact map graph are applied for smoothing before comparing the contact maps, resulting in a concordance score that can be used for quality control of biological replicates. We calculated the GenomeDISCO reproducibility score based on 40-kb resolution contact maps for the biological replicates of Hi-C libraries.

### Matrix resolution analysis

Matrix resolution analysis was performed using two methods. The first method was performed as described previously [8], in which the matrix resolution was defined as the smallest locus size at which 80% of loci showed at least 1000 contacts. The matrix resolution reflects the finest scale at which one can reliably discern local features. Using this method, we determined that the matrix resolution in this study was 10 kb.

The second method was to identify a “saturated” matrix resolution based on the valid pairs of Hi-C data of different soybean accessions. To this end, we first selected nine representative accessions with the highest, intermediate, and lowest numbers of valid pairs from the 27 soybean accessions, which included three wild soybeans, three landraces, and three improved cultivars. Next, we chose the number of valid pairs and the matrix resolution for a series of gradients. For the valid pair gradient, we randomly picked the same number of valid pairs from each representative accession. The initial number of valid pairs was 100,000,000, and the gradient was 40,000,000 until the number of valid pairs reached the total number of valid pairs of each accession. For the matrix resolution gradient, we chose a range of matrix resolutions: 5 kb, 10 kb, and 25 kb. We performed the evaluation of the valid pair gradient and the matrix resolution gradient with two methods: identification of TAD boundaries by insulation scores [16] method and identification of contact domains by Arrowhead [8] method. We determined that the insulation score method was relatively stable regardless of the valid pair gradient and the matrix resolution gradient, while the Arrowhead method reached saturation at a 25-kb matrix resolution for all representative accessions. Finally, we chose the robust resolution of 25 kb as the matrix resolution of the 27 soybean accessions.

### A/B compartment identification

E1 values from the eigenvector decomposition on Hi-C contact maps were used to indicate the A/B compartment status. We used cooltools software (v0.3.2) [74] with the parameter “cooltools call-compartments” to obtain the E1, E2, and E3 values based on a 100-kb resolution Hi-C contact matrix. Because E2 or E3 values sometimes reflect A/B compartments, we manually checked the E1, E2, and E3 tracks with gene density and the plaid pattern in the Hi-C contact maps along each chromosome and obtained the final “E1” list. The direction of the eigenvalues is arbitrary; therefore, negative values were set to “A,” and positive values were set to “B” based on their association with gene density. Compartment border was defined as the edge bin of the A/B compartments.

### A compartment percentage

A compartment percentage was defined as the percentage of the length of A compartment on one chromosome to the full length of the chromosome. In Fig. 1b, the average A compartment percentage was defined as the average of the A compartment percentages of 20 chromosomes in each soybean accession.

### I region identification

I regions were defined as the regions of the A compartment and B compartment intersection. To identify these regions in the genome, we used a 500-kb sliding window (5 bins) and a 100-kb step size (1 bin) to extract these compartment intersection regions. If a single window contained A compartment and B compartment at the same time, the bin was defined as an I region. For example, when we traced whether one bin belonged to the I region, we traversed the compartment status of this bin and the following four compartment bins. If there were both A and B compartments in the window (5 bins), we identified the bin as belonging to I regions.

### Pan-3D genome of A/B compartments

The pan-3D genome of the A/B compartments was based on the Reference mapping data, and A/B compartment identification was performed based on the ZH13 reference genome. We defined conservative compartments as compartment bins showing the same compartment status in all 27 accessions, which could be divided into three types: A compartments present in all 27 accessions, B compartments present in all 27 accessions, and NAs present in all 27 accessions. Similarly, we defined variable compartments as compartment bins that showed a different compartment status in at least one accession, which could be divided into three types: compartments with only A compartments (compartment bins containing only A compartments and NAs in all 27 accessions), compartments with only B compartments (compartment bins containing only B compartments and NAs in all 27 accessions), and compartments with both A and B compartments (AB variable compartments, compartment bins containing A and B compartments at the same time in all 27 accessions).

### Coefficient of compartment variation

The CCV was defined as the natural logarithm of the number of B compartments divided by the number of A compartments in the variable bin. The formula for calculating CCV was as follows:

$$\textrm{CCV}=\log \left(\#\textrm{B}/\#\textrm{A}\right)$$

For CCV calculation, we selected compartments with both A and B compartments (AB variable compartments).

As expected, the CCV could impact the size and orientation of compartment variation. The absolute value of the CCV reflected the variation size, such that the smaller the absolute value was, the greater the variation within the bin and, thus, the smaller the difference between the numbers of A compartments and B compartments within the bin; conversely, the greater the absolute value was, the lower the variation within the bin and, thus, the greater the difference between the numbers of A compartments and B compartments within the bin. A positive or negative CCV reflected the variation orientation: if the CCV value was positive, the number of B compartments was greater than the number of A compartments in the bin, and the bin thus tended toward a B compartment state; if the CCV value was negative, the number of A compartments was greater than the number of B compartments in the bin, and the bin thus tended toward an A compartment state.

### Genomic feature analysis

For GC content, we used bedtools software (v2.17.0) [75] with the option “nuc” to count the numbers of A, T, G, C, and N bases in the specific window of the soybean genome, and calculated the GC content of the window manually. For TSS density, we extracted all TSSs from gff3 files within the specific window of the soybean genome, after deduplication, we calculated the TSS density of the window and normalized it manually. For repeat percentage, we used the repeat datasets that we previously generated using repeatmasker [39] and calculated the repeat percentages in specific windows using our custom Perl script.

### TAD boundary identification

TAD boundaries were identified using the insulation score method [16] based on a 25-kb resolution KR normalized matrix with the parameters “--is 500000 --ids 250000.” This method first calculated the average number of contacts that occurred across each bin. This could be visualized by sliding a 500 kb × 500 kb (20 bins × 20 bins) square along the matrix diagonal and aggregating all signals within the square. The mean signal within the square was then assigned to the 25-kb diagonal bin, and this procedure was subsequently repeated for all 25-kb diagonal bins except bins within 500 kb of the matrix start/end. The insulation score was then normalized relative to all of the insulation scores across each chromosome by calculating the log2 ratio of each bin’s insulation score and the mean of all insulation scores. Valleys or minima along the normalized insulation score vector represented loci of reduced Hi-C interactions that occurred across the bin. These valleys or minima were interpreted as insulation boundaries or areas of high local insulation. The valleys or minima were identified as follows: first, a delta vector was calculated to approximate the slope of the normalized insulation vector. The delta vector was defined as the difference between the amount of the insulation change 125 kb to the left of the central bin and 125 kb to the right of the central bin. The delta vector crossed the horizontal 0 at all peaks and all valleys. All bins where the delta vector crossed 0 were extracted. Zero-crossings occurring at peaks were removed, and the remaining zero-crossings, all occurring at potential valleys, were passed through a boundary strength filter. The boundary strength was defined as the difference in the delta vector between the local maximum to the left and local minimum to the right of the boundary bin. All boundaries with a boundary strength < 0.1 were removed. TADs were identified as regions between two boundaries.

### Contact domain identification

Contact domains were identified with the Arrowhead algorithm using Juicer tools (v1.9.8) [71] based on a 25-kb resolution KR normalized matrix with the parameter “-r 25000.” Arrowhead is a heuristic algorithm for detecting the corners of domains to locate the boundaries of TADs. The algorithm first performed an “arrowhead” transformation on the normalized matrix, which is defined as *A*_*i,i+d*_ = (*M*_*i,i-d*_*– M*_*i,i+d*_)/(*M*_*i,i-d*_*+ M*_*i,i+d*_), where *M* is the normalized contact matrix and *A* is the arrowhead matrix. Based on this transformation, *A*_*i,i+d*_ will be strongly positive if locus *i* *− d* is inside a domain and locus *i + d* is not. If the reverse is true, *A*_*i,i+d*_ will be strongly negative. If the loci are both inside or both outside a domain, *A*_*i,i+d*_ will be close to zero. Consequently, each contact domain was replaced with an arrowhead-shaped motif after transformation and could be identified using dynamic programming.

### Directionality index calculation

The directionality index (DI) can measure the tendency of a locus to interact with upstream sites and downstream sites. The DI was calculated by domaincaller software [7] at 25-kb resolution contact maps and 2-Mb windows. It was calculated using the following equation: DI = ((*B* − *A*)/|*B* − *A*|)×((*A* − *E*)^2^/*E* + (*B* − *E*)^2^/*E*), where *A* was the number of reads that mapped from a given 25-kb bin to the upstream 2 Mb, *B* was the number of reads that mapped from the same 25-kb bin to the downstream 2 Mb, and *E* was the expected number of reads under the null hypothesis, which was equal to (*A* + *B*)/2.

### Repeat enrichment analysis

The repeat datasets of 27 soybean accessions were annotated as described above [39]. In our analysis, repeats were divided into 17 subtypes, including 14 subtypes of TEs and 3 subtypes of TRs. The TEs included DNA transposons (hAT, CACTA, Mutator, PIF/Harbinger, Tc1/Mariner, Tourist-like MITE, Stowaway-like MITE, Helitron, and unclassified DNA transposons), LTR retrotransposons (Gypsy, Copia, and unclassified LTR retrotransposons), and non-LTR retrotransposons (LINEs and SINEs). The TRs included simple repeats, low complexity repeats, and satellite repeats. We performed the repeat enrichment analysis of each subtype by calculating the percentages, numbers, and lengths of repeat fragments. For number and length calculations, we defined the repeat fragments within the bin based on the midpoint of the fragments.

### Genome coordinate conversion

To convert the genome coordinates of TAD boundaries between the ZH13 reference genome and the query genomes, we first generated custom UCSC chain files between the reference genome and query genomes using the UCSC liftOver tool (http://genome.ucsc.edu). Thereafter, we performed genome coordinate conversion using CrossMap (v0.2.9) [76] with the option “CrossMap.py region.”

### Comparative 3D genome of TAD boundaries

After genome coordinate conversion, we performed the pairwise comparison of the TAD boundaries of 26 query accessions with the TAD boundaries of reference accession ZH13 in the reference genome. Considering the bias in the identification of boundaries, we allowed these boundaries to be shifted by up to 25 kb (1 bin) in each flank. This meant that the 75-kb (3 bins) boundary regions were compared between reference accession ZH13 and the other 26 query accessions. If there was overlap between the reference boundary region and the query boundary region, we considered them to be matched; otherwise, we considered them to be mismatched. The matched boundaries of the reference accession were referred to as reference-common boundaries, and the mismatched boundaries of the reference accession were referred to as reference-specific boundaries. Analogously, the matched boundaries of the query accessions were referred to as query-common boundaries, and the mismatched boundaries of the query accessions and those boundaries that could not be translated to the reference genome via genome coordinate conversion were collectively referred to as query-specific boundaries. The comparisons were performed individually for each of the 26 query accessions.

### Pan-3D genome of TAD boundaries

#### Sequence extraction

To increase the robustness of TAD boundary identification, we extracted the boundaries as well as the flanking 25-kb (1 bin) regions on both sides of the boundaries. This 75-kb (3 bins) region was defined as the boundary region of each boundary.

#### Pairwise alignment

To evaluate the similarity of each accession’s boundary regions, we performed pairwise alignment using the sequence alignment software MUMmer4 [77]. The alignment was performed by using the “nucmer” command with the parameters “-c 5000 -b 1000,” and filtering was performed by using “delta-filter” with the default parameters and the “show-coords” with parameters “-bcHlorT”. Alignment between two accessions was performed in the forward and reverse directions at the same time. Consequently, the total number of alignments was 729 (27^2 ^= 729).

#### Similarity statistics

After alignment, we calculated the sequence similarity of boundary regions. Similarity was calculated as the percentage of the mapped sequence to the full length of the boundary regions. Consequently, we considered two boundary regions to be the same boundary region based on the similarity of the mapped sequences of the two boundary regions.

#### Boundary clustering

Based on the similarity evaluation of boundary regions, we clustered the matching boundary regions. Clustering was performed using the 729 similarity files by our custom clustering script. Finally, we obtained the clustering results of all boundary regions of the 27 accessions.

#### Pan-3D genome

The final pan-3D genome was presented in files with two different formats: the first file was in csv format, and the second was in table format.

### GO enrichment analysis

The GO term annotation of the protein sequences encoded by the transcripts of the 27 soybean accessions was performed using PANNZER2 [78]. Enrichment analysis was performed using the R package clusterProfiler (v3.10.1) [79]. Only statistically significant (FDR < 0.01) GO terms were used.

### SV datasets

The SV datasets of the ZH13 soybean accession and 26 other soybean accessions were generated from our previous pan-genome analyses of wild and cultivated soybeans [39]. For PAV identification, we used SVMU to identify these variations based on the results provided by NUCmer. For CNV identification, we filtered synteny blocks of less than 100 bp in the results of MUMmer4 and identified CNVs from regions with two or more overlapping synteny blocks (>90% identity). INV and TRANS were identified by manual checking depending on their locations and orientations with respect to neighboring blocks according to the nonallelic homology blocks.

### SV distribution in A/B compartments

For A/B compartments, we investigated the distribution of observed and expected values of SVs in the A compartments, where expected values were generated by the bootstrapping method of generating random A compartments 10,000 times in the genome. We calculated the numbers of SVs within A compartments and then calculated *z* scores and *P* values. Additionally, we observed the distribution of the four types of SVs in I regions using the same method described above. In this study, we analyzed each PAV, INV, CNV, and TRANS event independently, although these SVs might occur concurrently in a given sample.

### SV distribution around TAD boundaries

For TAD boundaries, we calculated the percentage of four types of SVs around boundaries. This analysis was performed based on the comparative 3D genome and pan-3D genome of TAD boundaries.

### SV classification

We classified the SVs according to the positional relationship between SVs and TAD boundaries. In brief, we divided SVs into three types: ABA SVs were defined as SVs spanning the whole length of one boundary, PBA SVs were defined as SVs spanning part of the length of one boundary, and NBA SVs were defined as SVs within TADs.

### SV effects and contributions

To identify SV effects on and contributions to TAD boundary variation, we performed association analysis of SVs and comparative 3D genome of TAD boundaries. For each of the eight types of SVs (ABA-PAV, PBA-PAV, ABA-CNV, PBA-CNV, ABA-INV, PBA-INV, ABA-TRANS, and PBA-TRANS), we classified the boundaries into four types: common boundaries with SVs, common boundaries without SVs, specific boundaries with SVs, and specific boundaries without SVs. The contribution of each SV type is defined as the proportion of specific boundaries that can be explained by each type. The effect size of each SV type is defined as the proportion of specific boundaries with the SV relative to all the boundaries with the SV.

### RNA-seq data processing

#### Read alignment and quantification

Clean reads were mapped to the genome of each accession by using HISAT2 (v2.1.0) with the default parameters [80]. An annotation file in GFF3 format was provided to StringTie (v1.3.4) using the -G option for the transcript assembly process [81]. Fragments per kilobase of transcript per million reads mapped (FPKM) values were also calculated by StringTie software.

#### Differential gene expression analysis

For differential expression analysis, clean reads were mapped to the ZH13 genome by HISAT2 with the default parameters. Gene expression FPKM values were calculated by StringTie software as described previously. Differential gene expression was performed using DESeq2 (v1.4.5) with the default parameters [82]. FDR <0.05 and |fold change| > 2 were used as cutoffs for significantly differentially expressed genes.

### Enrichment of DEGs

To analyze the density of DEGs, we enriched all the DEGs around TAD boundaries and calculated their average number as the DEG density. For the enrichment of the comparative 3D genome and pan-3D genome of TAD boundaries, we performed the analysis in 26 query accessions one by one and averaged the results.

### NonVariation-gene analysis

NonVariation-genes were defined as genes that had no variations in reference accession and query accession. We used genome variation data, which included single-nucleotide variants (SNVs), insertions and deletions (InDels), and SVs from the de novo genome assembly, to identify nonVariation-genes.

### Genome scanning for selective signals

We performed a genome cross-population *F*_*ST*_ value scan using the whole-genome sequencing (WGS) data from 2898 soybean accessions [39]. *F*_*ST*_ values were calculated with a 25-kb window using VCFtools. Evidence for selection across the genome during domestication and improvement was evaluated in two comparisons: landraces versus *G. soja* for domestication and improved cultivars versus landraces for improvement. The highest *F*_*ST*_ values, accounting for 5% of the genome, were considered as selected regions.

### Quantitation and statistical analysis

The R language was used for statistical analysis. The Wilcoxon rank-sum test was used for the assessment of statistical significance with the wilcox.test function in R. The Kolmogorov–Smirnov test was used to calculate statistical significance for the difference between densities of boundaries in the A and B compartments. Fisher’s exact test was used for statistical significance with the fisher.test function in R. * indicates *P* < 0.05, ** indicates *P* < 0.01, *** indicates *P* < 0.001; n.s. indicates *P* > 0.05.

## Supplementary Information

**Additional file 1: Figure S1.** Quality metrics and repeatability of sequencing datasets. **Figure S2.** Identification of I regions. **Figure S3.** Conservative and variable compartments analysis. **Figure S4.** TAD boundary statistics and TE enrichment in 27 soybean accessions. **Figure S5.** Comparative 3D genome and pan-3D genome of TAD boundaries. **Figure S6.** Structural variation analysis of the 3D genome in 27 soybean accessions. **Figure S7.** Expression analysis of A/B compartments. **Figure S8.** Expression analysis of TAD boundaries. **Figure S9.** Examples of expression variations related to TAD boundaries. **Figure S10.** 3D genome dynamics during domestication and improvement.

**Additional file 2: Table S1.** Self mapping and Reference mapping statistics of Hi-C data. **Table S2.** A/B compartment identification in 27 soybean accessions. **Table S3.** Pan-3D genome of A/B compartments. **Table S4.** TAD boundary identification in 27 soybean accessions. **Table S5.** Pan-3D genome of TAD boundaries. **Table S6.** nonVariation-DEGs of soybean accessions.

**Additional file 3.** Review history.

## Data Availability

The sequencing data generated in this study are available at the Genome Sequence Archive (GSA, https://bigd.big.ac.cn/gsa/; accession number: PRJCA009364) [83]. Custom UCSC chain files between ZH13 and 26 query genomes: https://figshare.com/articles/dataset/UCSC_chain_files_of_soybean_genomes/20027336 [84]. Computational scripts used for data analyses conducted as part of this study are available under MIT license at Github: https://github.com/LingbinNi/soybean_pan-3D_genome_analysis [85] and Zenodo: 10.5281/zenodo.7514511 [86].
