# Supplementary material for: Pan-3D genome analysis reveals structural and functional differentiation of soybean genomes
Source: Genome Biol. 2023 Jan 19;24:12. doi: 10.1186/s13059-023-02854-8 (PMC9850592; doi:10.1186/s13059-023-02854-8)
Supplement: Supplementary file 1 — Additional file 1: Figure S1. Quality metrics and repeatability of sequencing datasets. Figure S2. Identification of I regions. Figure S3. Conservative and variable compartments analysis. Figure S4. TAD boundary statistics and TE enrichment in 27 soybean accessions. Figure S5. Comparative 3D genome and pan-3D genome of TAD boundaries. Figure S6. Structural variation analysis of the 3D genome in 27 soybean accessions. Figure S7. Expression analysis of A/B compartments. Figure S8. Expression analysis of TAD boundaries. Figure S9. Examples of expression variations related to TAD boundaries. Figure S10. 3D genome dynamics during domestication and improvement. [file 13059_2023_2854_MOESM1_ESM.pdf]

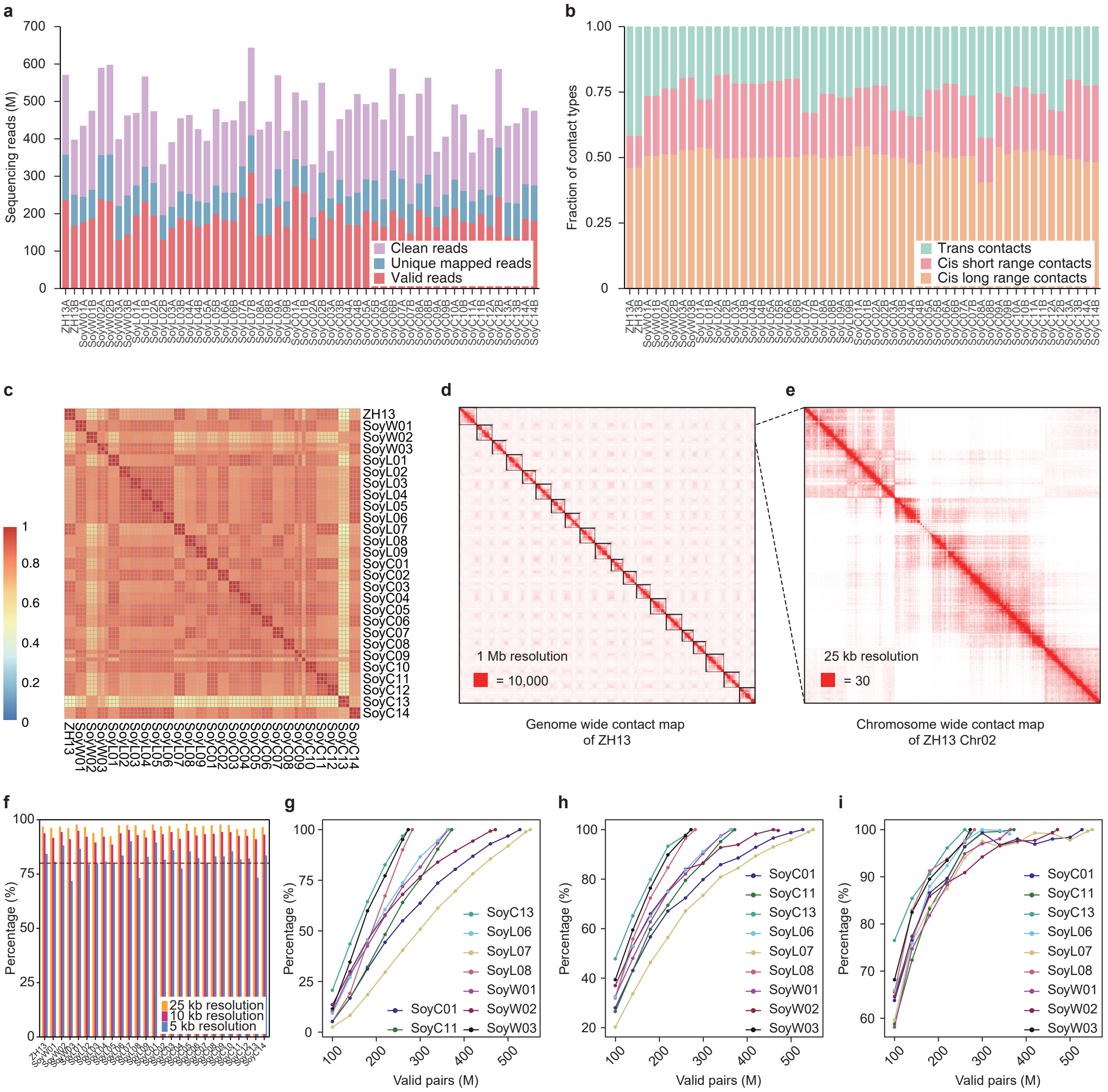

Fig. S1 Quality metrics and repeatability of sequencing datasets. a Clean reads, unique mapped reads and valid reads statistics of the self mapping results of each Hi-C library. **b** Fraction of contact types of cis (intra-chromosome) contacts and trans (inter-chromosome) contacts of each Hi-C library. The cis short range contacts represent cis contacts with the contact distance of no more than 20 kb, while the cis long range contacts represent cis contacts with the contact distance greater than 20 kb. **c** GenomeDISCO repeatability scores among the Hi-C data of replicate 1, replicate 2 and pooled library of each accession. **d** Genome-wide contact map of ZH13. The black boxes represent Hi-C contacts within chromosomes. **e** Chromosome-wide contact map of Chr02 of ZH13. **f** Matrix resolution analysis using the method that 80% of loci have at least 1,000 contacts. **g** Percentages of the number of contact domains identified relative to the maximum number of contact domains identified in nine selected accessions at 5 kb resolution. **h** Percentages of the number of contact domains identified relative to the maximum number of contact domains identified in nine selected accessions at 10 kb resolution. **i** Percentages of the number of contact domains identified relative to the maximum number of contact domains identified in nine selected accessions at 25 kb resolution.

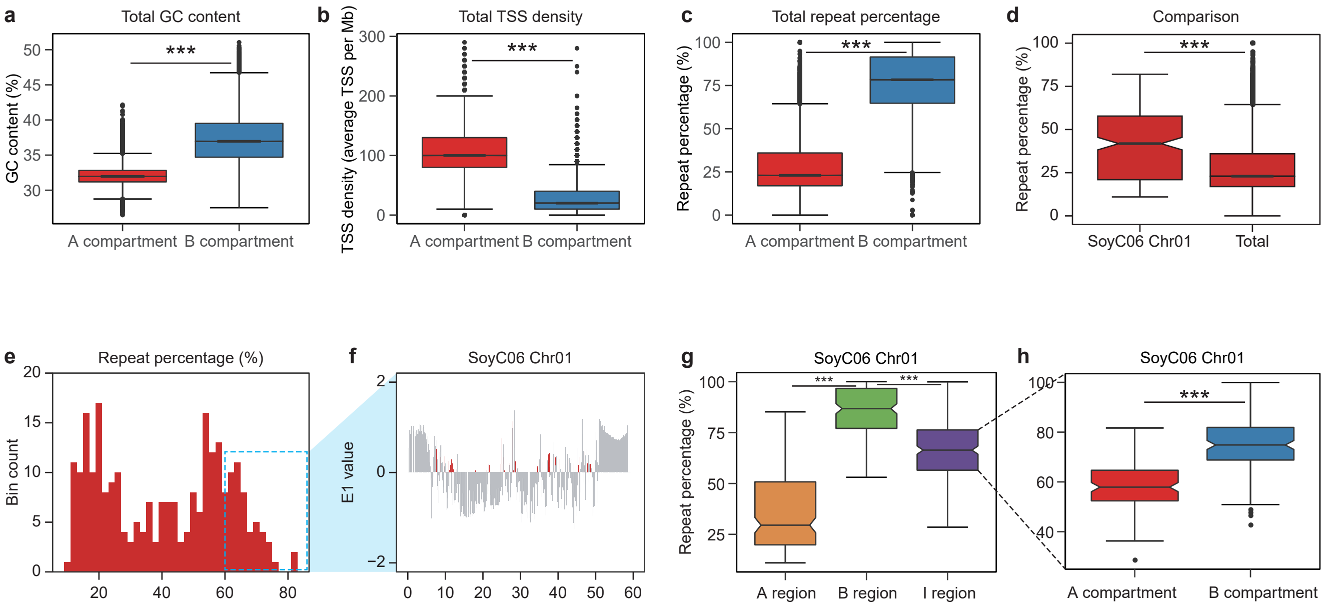

Fig. S2 Identification of I regions. a Box plot of the GC contents of the A compartments and B compartments of 27 soybean accessions. **b** Box plot of the TSS densities in the A compartments and B compartments of 27 soybean accessions. **c** Box plot of the repeat percentage of the A compartments and B compartments of 27 soybean accessions. **d** Box plot of the repeat percentages of the A compartments of SoyC06 chromosome 1 and 27 soybean accessions. **e** Distribution of the repeat percentages in individual bins of the A compartment of chromosome 1 in SoyC06. **f** Positions of high-repeat-percentage (repeat percentage >= 60%) bins of A compartment of chromosome 1 in SoyC06. **g** Box plot of the repeat percentages of the A regions, B regions and I regions of chromosome 1 in SoyC06. **h** Box plot of the repeat percentages of the A compartments and B compartments in the I regions of chromosome 1 in SoyC06. In (a–d), (g) and (h), the boxes represent the 25th, 50th and 75th percentiles, and whiskers represent 1.5× the interquartile range. ***$P < 0.001$ for pairwise comparisons between two types (two-sided Wilcoxon rank-sum test).

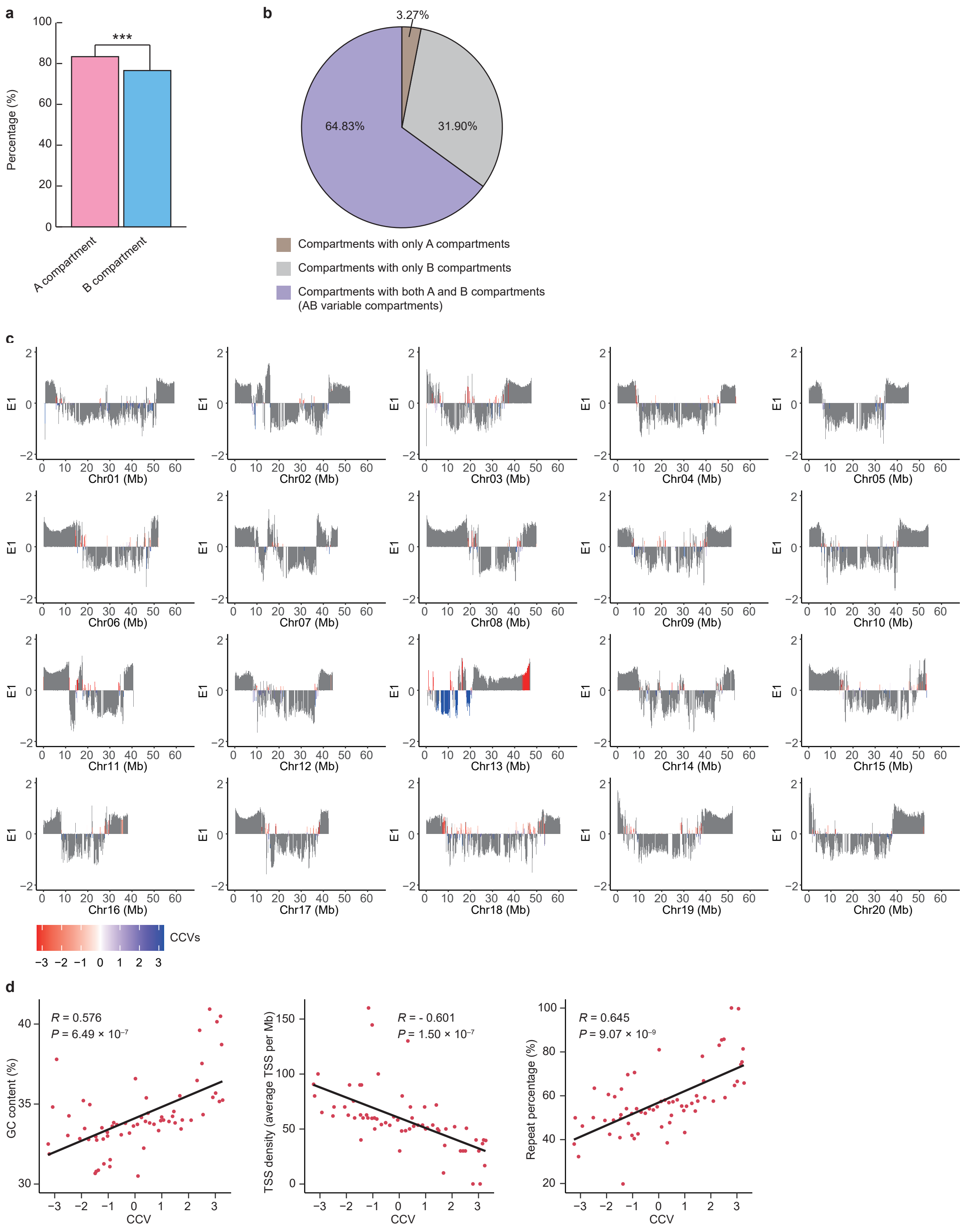

Fig. S3 Conservative and variable compartments analysis. a Percentage of conservative A compartments among all A compartments and conservative B compartments among all B compartments. ****P* < 0.001 for pairwise comparison between two types (two-sided Fisher's exact test). **b** Pie chart of compartment with only A compartments, compartment with only B compartments and AB variable compartments. **c** AB variable compartments with CCVs. Gray compartments represent non-AB variable compartments. **d** Correlation analysis of CCVs and average GC contents, TSS densities and repeat percentages.

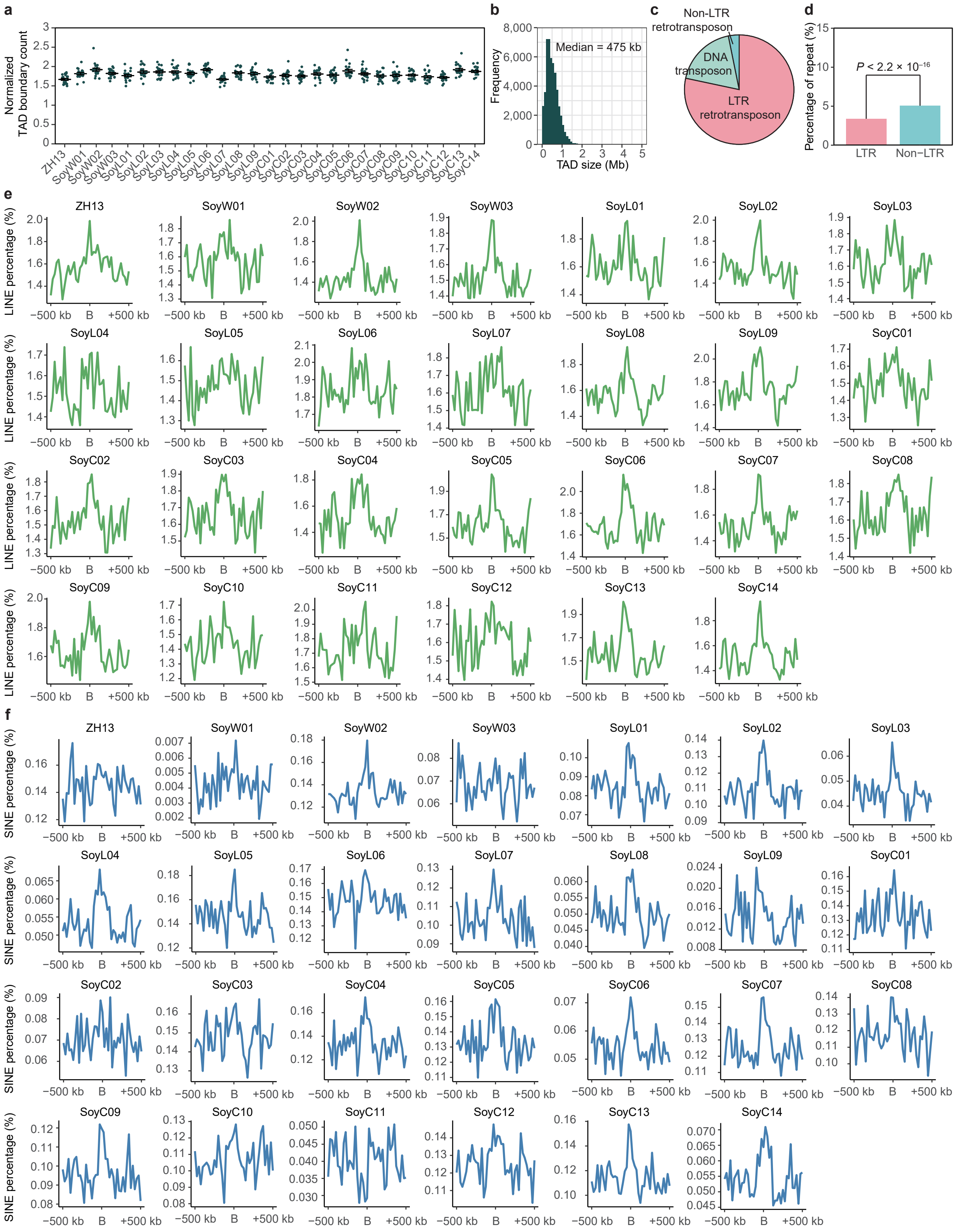

Fig. S4 TAD boundary statistics and TE enrichment in 27 soybean accessions. a Normalized TAD boundary count (average TAD boundary number per Mb) on 20 chromosomes of 27 soybean accessions. Each dot represents one chromosome. Data are mean ± s.e.m. **b** Histogram of TAD sizes in 27 soybean accessions. The median TAD size is shown. **c** Relative percentage of transposons in the ZH13 accession. **d** LTR retrotransposon and non-LTR retrotransposon enrichment of TAD boundaries in the ZH13 accession. *P* value was calculated by two-sided Fisher's exact test. **e** Enrichment of LINE elements around TAD boundaries in individual accessions. B, boundary. **f** Enrichment of SINE elements around TAD boundaries in individual accessions. B, boundary.

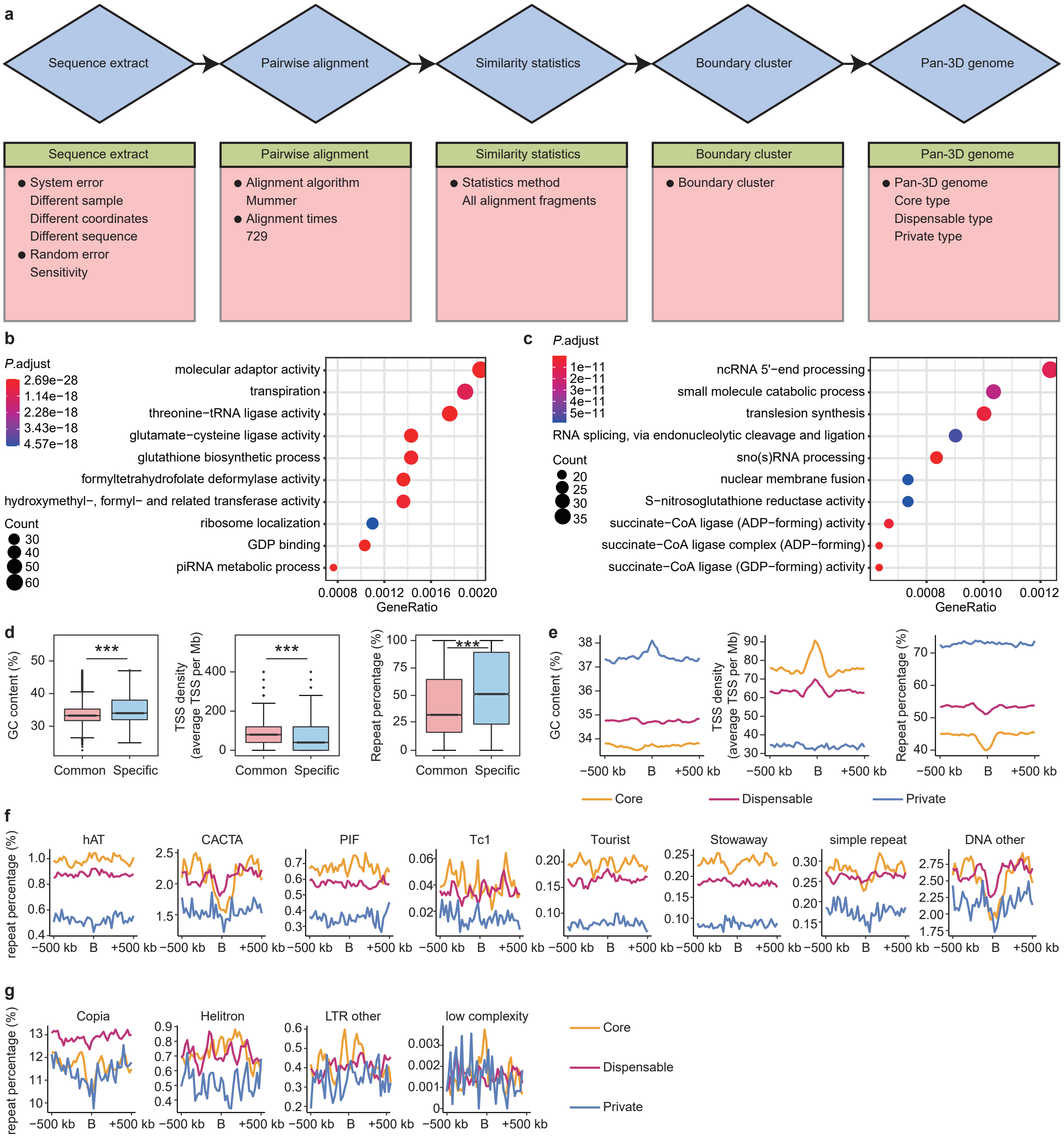

Fig. S5 Comparative 3D genome and pan-3D genome of TAD boundaries. **a** Pan-3D genome workflow. **b** GO analysis of core TAD boundaries. **c** GO analysis of dispensable TAD boundaries. **d** Box plot of the GC contents, TSS densities and repeat percentages of common and specific TAD boundaries. ****P* < 0.001 for pairwise comparisons between two types (two-sided Wilcoxon rank-sum test). **e** Enrichment of the GC contents, TSS densities and repeat percentages of core, dispensable and private TAD boundaries of 27 soybean accessions. **f** Enrichment of Type Ⅱ repeats at the core, dispensable and private TAD boundaries of 27 soybean accessions. **g** Enrichment of uncertain repeats at the core, dispensable and private TAD boundaries of 27 soybean accessions.

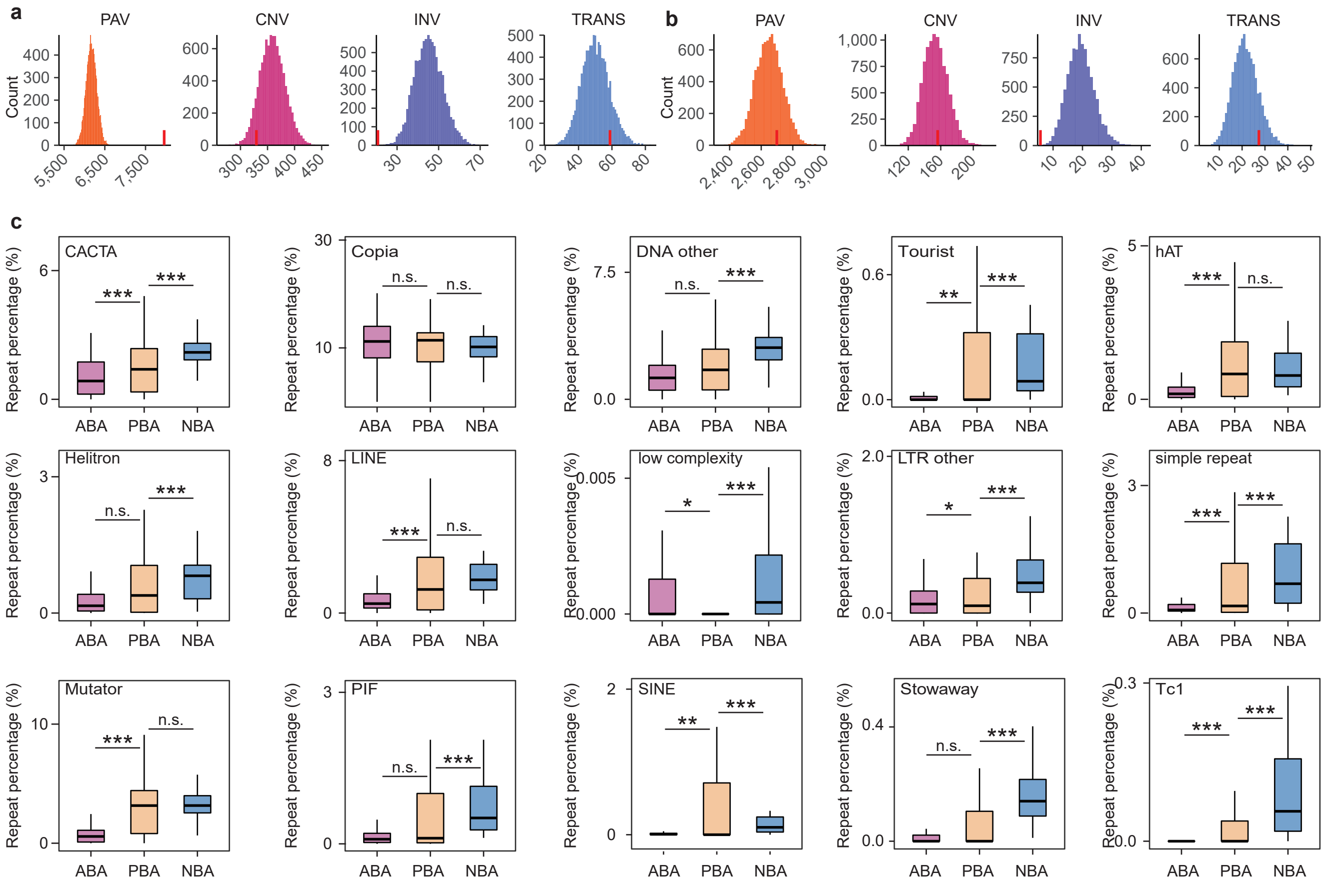

Fig. S6 Structural variation analysis of the 3D genome in 27 soybean accessions. a Observed (red bar) and expected distributions (histograms) of PAV, INV, CNV and TRANS of A compartments in ZH13. The expected distribution is based on compartment data randomly shuffled by bootstrapping (n = 10,000). **b** Observed (red bar) and expected distributions (histograms) of PAV, INV, CNV and TRANS of I regions in ZH13. The expected distribution is based on compartment data randomly shuffled by bootstrapping (n = 10,000). **c** Percentage of repeat elements of ABA, PBA and NBA SVs. Adjusted P values were calculated by two-sided Wilcoxon rank-sum test with Benjamini–Hochberg multiple testing correction. *$P < 0.05$, **$P < 0.01$, ***$P < 0.001$; n.s., not significant.

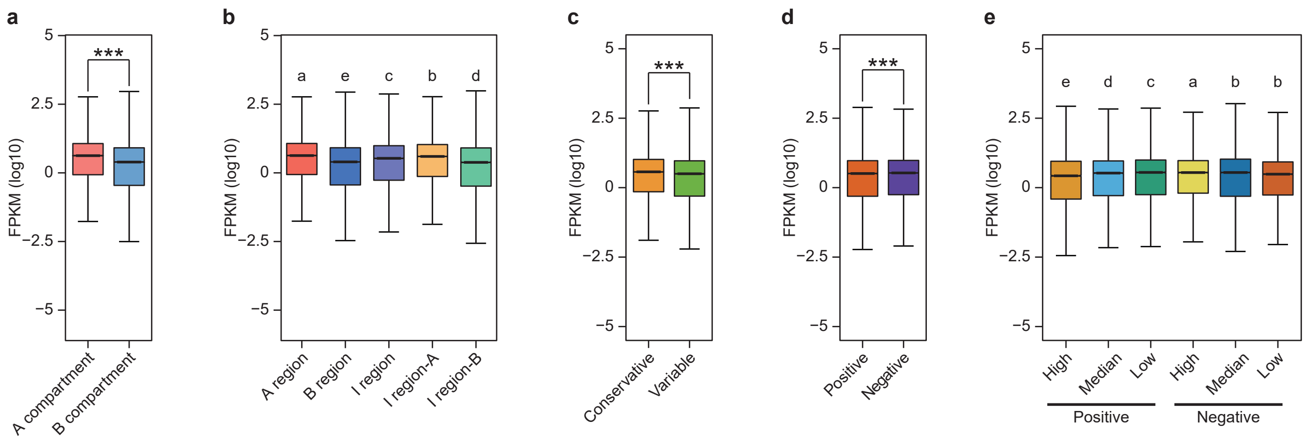

Fig. S7 Expression analysis of A/B compartments. a Expression of A compartments and B compartments in 27 soybean accessions. ***P < 0.001 for pairwise comparisons between two types (two-sided Wilcoxon rank-sum test). **b** Expression of A regions, B regions, I regions, I regions-A (A compartments in I regions) and I regions-B (B compartments in I regions) in 27 soybean accessions. Multiple comparisons were performed by one-sided Wilcoxon rank-sum test with Benjamini–Hochberg multiple testing correction. **c** Expression of conservative and variable compartments. ***P < 0.001 for pairwise comparisons between two types (two-sided Wilcoxon rank-sum test). **d** Expression of AB variable compartments with positive and negative CCVs. ***P < 0.001 for pairwise comparisons between two types (two-sided Wilcoxon rank-sum test). **e** Expression of AB variable compartments with high (highest 30%), median (median 40%), and low (lowest 30%) absolute values of positive and negative CCVs. Multiple comparisons were performed by one-sided Wilcoxon rank-sum test with Benjamini–Hochberg multiple testing correction. In (**a**–**e**), FPKM, fragments per kilobase of transcript per million reads mapped.

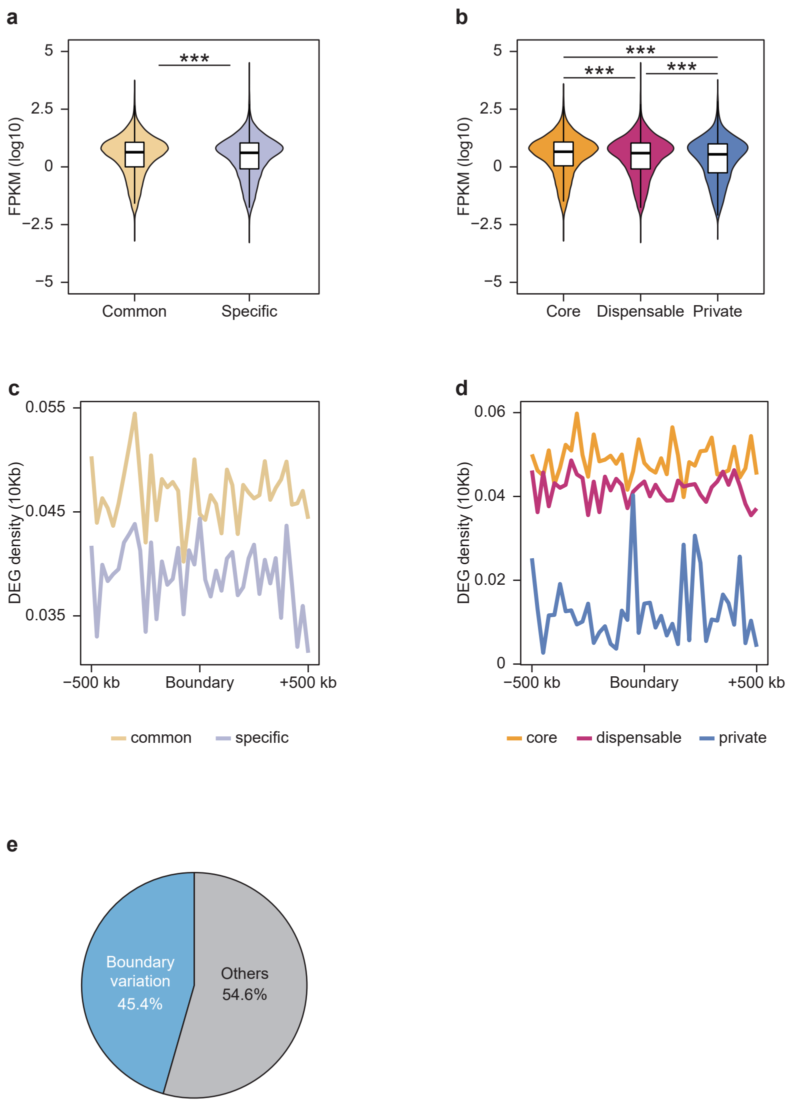

Fig. S8 Expression analysis of TAD boundaries. a Expression of common and specific TAD boundaries in the comparative 3D genome. ****P* < 0.001 for pairwise comparisons between the two types (two-sided Wilcoxon rank-sum test). **b** Expression of core, dispensable and private TAD boundaries in the pan-3D genome. ***Adjust *P* < 0.001 for multiple comparisons (one-sided Wilcoxon rank-sum test with Benjamini–Hochberg multiple testing correction). **c** DEG density around common and specific TAD boundaries. **d** DEG density around core, dispensable and private TAD boundaries. **e** Pie chart of nonVariation-DEGs that can be explained by TAD boundary effects. In (**a**–**b**), FPKM, fragments per kilobase of transcript per million reads mapped. In (**c**–**d**), DEG, differentially expressed gene.

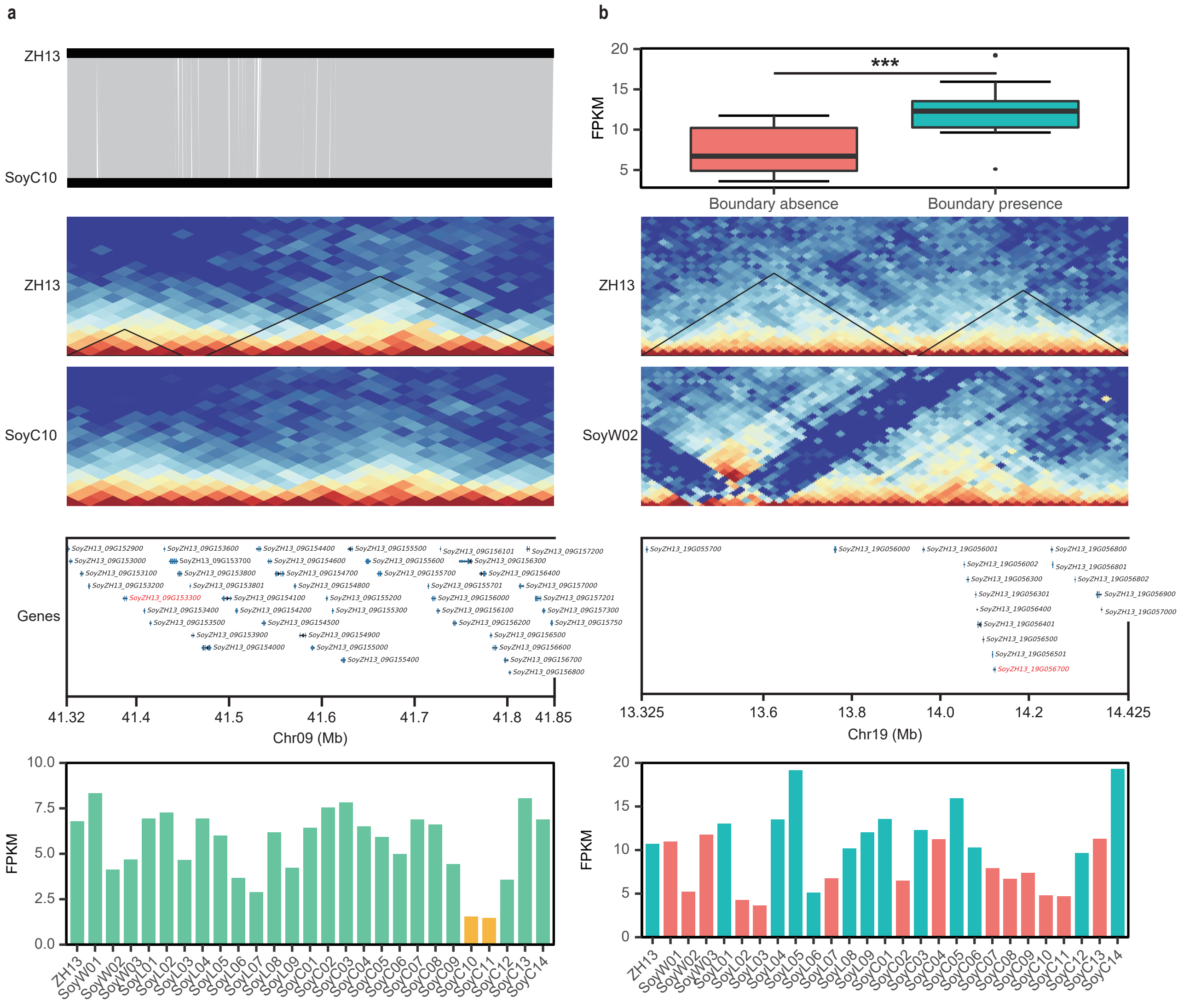

Fig. S9 Examples of expression variations related to TAD boundaries. a Genome synteny analysis, Hi-C contact maps and gene panels in Chr09: 41,320,000-41,850,000. The barplot at the bottom shows the expression levels of *SoyZH13_09G153300*. **b** Gene expression of two groups, Hi-C contact maps and gene panels in Chr19: 13,325,000-14,325,000. The barplot at the bottom shows the expression levels of *SoyZH13_19G056700. P* value is calculated by one-sided Wilcoxon rank-sum test.

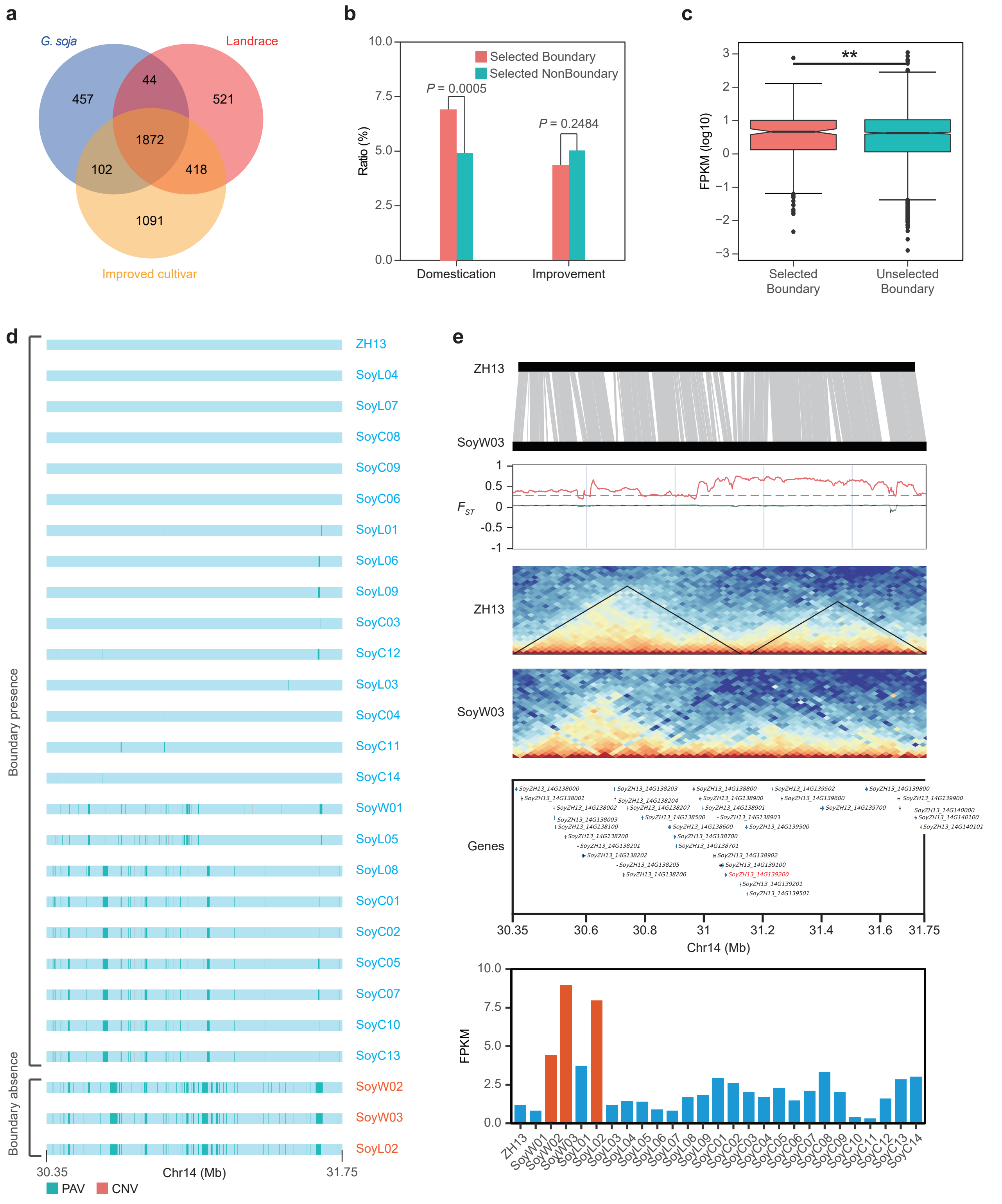

Fig. S10 3D genome dynamics during domestication and improvement. a Venn plot of TAD boundaries in *G. soja*, landraces and improved cultivars. **b** Ratio of selected boundary regions and non-boundary regions during domestication and improvement. *P* value is calculated by one-sided Fisher's exact test. **c** Gene expression of selected boundaries and unselected boundaries. *P* value is calculated by one-sided Wilcoxon rank-sum test. **d** Overview of genomic SVs in Chr14: 30,350,000-31,750,000. **e** Genome synteny analysis, selection signals, Hi-C contact maps and gene panels in Chr09: 41,320,000-41,850,000. The selection signals during domestication is depicted in red line and the selection signals during improvement is depicted in green line in the selection signals panel. The red dashed line represents thresholds during domestication and green dashed line represents thresholds during improvement. The barplot at the bottom shows the expression levels of *SoyZH13_14G139200*.
