# Supplementary material for: Pan-3D genome analysis reveals structural and functional differentiation of soybean genomes
Source: Genome Biol. 2023 Jan 19;24:12. doi: 10.1186/s13059-023-02854-8 (PMC9850592; doi:10.1186/s13059-023-02854-8)
Supplement: Supplementary file 3 — Additional file 3. Review history. [file 13059_2023_2854_MOESM3_ESM.docx]

Review History

**First round of review**

**Reviewer 1**

**Were you able to assess all statistics in the manuscript, including the appropriateness of statistical tests used?**

Yes, and I have assessed the statistics in my report.

**Comments to author:**

The authors performed a pan-3D genome analysis using Hi-C data from 27 soybean accessions. Most of the results are about the statistical analysis of the data, and some of the analysis is a replication of known findings. There are fewer results for in-depth investigations of specific biological questions. In the Conclusion section, the authors claim that this study may provide another solution for plant breeding by modifying the 3D genome, however, no new concepts or related results are provided. Other suggestions are shown below.

1. The GenomeDISCO is a good way used to assess the repeatability between two Hi-C libraries. All the Hi-C libraries needed to calculate the repeatability score, not only for ZH13. I suggest using heatmaps to show this result.

2. The method about the calculation of Hi-C library resolution is: "We define the 'matrix resolution' of a Hi-C map as the locus size used to construct a particular contact matrix and the 'map resolution' as the smallest locus size such that 80% of loci have at least 1,000 contacts." in the reference (Rao et al., 2014). In my opinion, Figure S1f-i can not illustrate the saturation of the 3D chromatin structure that occurred in a 25 kb contact map.

3. Chromosome start and end positions should be labeled in Figure 1a. The pattern of A/B compartment is different from that in other plants, and the authors may reverse the bar-plots.

4. How to calculate the average A compartment percentages in Figure 1b? I can not find it in the methods.

5. In PAGE 5, LINE 40-44, "A compartment on some chromosomes in some accessions was significantly higher than the average level, as observed on chromosome 1 of SoyC06 (Additional file 1: Fig. S2d)." But Figure S2d shows us the comparison between A and B compartments.

6. How to determine that "I region" is not linked to the quality of Hi-C library data? The number of I regions should be shown in Figure 1d.

7. Coefficient of variation means that SD/Mean (Standard deviation/Mean). The CCV (compartment coefficient of variation) was defined as the natural logarithm of the number of B compartments divided by the number of A compartments in AB variable compartment bins. In PAGE 6, LINE 44-50, I think the CCV does not indicate a coefficient of variation. I suggest using other abbreviations to describe the algorithm.

8. Because the CCV is defined as B compartment divided by A compartment, it is positively correlated with E1. If CCV means B compartment divided by A compartment, the relation is negative. So I do not know why the author considers this result is "interesting"? In PAGE 6, in line 52.

9. Why two lines of insulation score are shown in figure 3a? What does each of them mean?

10. I do not know how to identify the compartment border in LINE 56-58.

11. In the comparative 3D genome of TAD boundaries sections, how to convert the genome coordinate in page 21?

12. In page 22, what clustering algorithm was used for boundary clustering?

13. "Although some SVs may not change the properties of A/B compartments, they affected the observed E1 values, such as the two large translocations on chromosome 11 and chromosome 13 in SoyW02 (Fig. 5b)", in PAGE 10. This conclusion needs statistic results to illustrate.

14. Which 3D genomic structures are conserved and which are specific between wild soybeans, landraces, and improved cultivars?

15. The relationship between 3D genome variation and gene expression is an interesting scientific question. I would like to have a more in-depth study that does not stop at the level of statistical analysis of data and replication of known results but is preferably related to specific biological questions.

16. The authors construct 3D genome maps for wild soybean, landraces and improved cultivars, but did not perform comparison between these groups. I suggest they perform this analysis to explore the changes of 3D genome organization during domestication.

**Reviewer 2**

**Were you able to assess all statistics in the manuscript, including the appropriateness of statistical tests used?**

Yes, and I have assessed the statistics in my report.

**Comments to author:**

In a previous work, the authors generated a pan-genome resource of soybean. In this work, the authors presented pan-3D genome of soybean using Hi-C sequencing. Since the diverse accessions had high-quality genome assemblies, the relationship between 3D genomic variation and structural variations were well analyzed. Besides, in-depth analyses enabled the finding of the links between 3D variation and gene expressions. Generally, this is a very nice work and provides an important resource for further investigations.

Below are several minor suggestions for the manuscript:

1. The Abstract of the manuscript should be rewritten, because many important results (e.g., the links between 3D variation and gene expressions) were not included.

2. Page 5 Line 41, "some chromosomes in some accessions". The fuzzy descriptions should be revised.

3. Page 8, Line 2, "Fisher's exact test, p < 2.2 × 10-16". 2.2 × 10-16 is a threshold? It should be noted in the main text. Moreover, p should be italic.

4. More gene-level examples, especially the well-known genes in soybean, could be added in the section of "3D genome variation and gene expression" and "Effect and contribution of SVs to 3D genome variations".

**Authors Response**

**Point-by-point responses to the reviewers’ comments:**

Reviewer #1:

The authors performed a pan-3D genome analysis using Hi-C data from 27 soybean accessions. Most of the results are about the statistical analysis of the data, and some of the analysis is a replication of known findings. There are fewer results for in-depth investigations of specific biological questions. In the Conclusion section, the authors claim that this study may provide another solution for plant breeding by modifying the 3D genome, however, no new concepts or related results are provided. Other suggestions are shown below.

*【Response】We thank you so much for the constructive suggestions, which help us to improve the manuscript significantly. We have carefully revised the manuscript and added more analyses. We hope the revised manuscript will meet your requirement.*

1. The GenomeDISCO is a good way used to assess the repeatability between two Hi-C libraries. All the Hi-C libraries needed to calculate the repeatability score, not only for ZH13. I suggest using heatmaps to show this result.

*【Response】Appreciate you so much for this constructive comment and also give us such good solution to show the results. We have added the results in the Fig. S1c.*

2. The method about the calculation of Hi-C library resolution is: "We define the 'matrix resolution' of a Hi-C map as the locus size used to construct a particular contact matrix and the 'map resolution' as the smallest locus size such that 80% of loci have at least 1,000 contacts." in the reference (Rao et al., 2014). In my opinion, Figure S1f-i can not illustrate the saturation of the 3D chromatin structure that occurred in a 25 kb contact map.

*【Response】We apologize that we did not illustrate the results clearly.*

*1) We initially used the method of Rao et al. to analyze the 'matrix resolution', and found that the resolution of each accession’s contact matrix can reach 10 kb. We have changed the original Fig. S1f as following figure.*

*2) In this study, our purpose is to compare the 3D genomic structures in different accessions and construct the pan-3D genome. To make the comparison strict, determining a ‘saturation’ resolution in 3D genomic structure identification for all accessions is very necessary. Therefore, we used the ‘gradient valid pair gradient resolution method’ to analyze contact matrix resolution again. Based on the investigation of 5 kb, 10 kb, and 25 kb resolutions, we found that at the resolution of 25 kb, most of the accession reach to saturation. Therefore, we determine the resolution as 25 kb.*

3. Chromosome start and end positions should be labeled in Figure 1a. The pattern of A/B compartment is different from that in other plants, and the authors may reverse the bar-plots.

*【Response】Thank you so much for the suggestions. We have labeled the start and end positions of chromosomes in Fig. 1a.*

*In previous studies, some researchers used negative values as A compartments, for example, in a study on rice (Liu et al., 2017), and some researchers used positive values as A compartments, for example, in a study on cotton (Wang et al., 2018). Therefore, we previously took negative values as the eigenvector values of A compartments. Following your suggestion, we have changed A compartments to positive eigenvalues throughout the manuscript.*

*References*

*Liu, C., Cheng, Y.J., Wang, J.W., and Weigel, D. (2017). Prominent topologically associated domains differentiate global chromatin packing in rice from Arabidopsis. Nat Plants 3, 742-748.*

*Wang, M., Wang, P., Lin, M., Ye, Z., Li, G., Tu, L., Shen, C., Li, J., Yang, Q., and Zhang, X. (2018). Evolutionary dynamics of 3D genome architecture following polyploidization in cotton. Nat Plants 4, 90-97.*

4. How to calculate the average A compartment percentages in Figure 1b? I can not find it in the methods.

*【Response】Thank you for the suggestion. The calculation was described in the figure legend “A compartment percentages across chromosomes in 27 soybean accessions. Each dot represents one chromosome. Data are mean ± s.d”. Taking your advice, we have added a detailed description in the methods.*

*“A compartment percentage*

*A compartment percentage was defined as the percentage of the length of A compartment on one chromosome to the full length of the chromosome. In Fig. 1b, the average A compartment percentage was defined as the average of the A compartment percentages of 20 chromosomes in each soybean accession.”*

5. In PAGE 5, LINE 40-44, "A compartment on some chromosomes in some accessions was significantly higher than the average level, as observed on chromosome 1 of SoyC06 (Additional file 1: Fig. S2d)." But Figure S2d shows us the comparison between A and B compartments.

*【Response】Thank you for the comment. We have revised the text and replaced the Fig. S2d. “Nevertheless, we surprisingly found that the repeat percentage of the A compartment on several chromosomes was significantly higher than the average level, such as chromosome 1 of SoyC06 (Additional file 1: Fig. S2d).”*

6. How to determine that "I region" is not linked to the quality of Hi-C library data? The number of I regions should be shown in Figure 1d.

*【Response】Thank you so much for this concern and good suggestion.*

*We agree with you that I region may be linked to the quality of Hi-C library data. Evaluation of the data quality determined that the quality from this study is in a high quality and is comparable to the published data either from soybean or from other plants. In fact, this phenomenon was also found in animal (Johnstone et al., 2020). Therefore, we believe I region is commonly existed in the genome. However, along with the technology development, higher quality Hi-C data may help us to reduce the analysis error and reveal more interesting discoveries.*

*References*

*Johnstone, S.E., Reyes, A., Qi, Y., Adriaens, C., Hegazi, E., Pelka, K., Chen, J.H., Zou, L.S., Drier, Y., Hecht, V., et al. (2020). Large-Scale Topological Changes Restrain Malignant Progression in Colorectal Cancer. Cell 182, 1474-1489 e1423.*

*Following your suggestion, we have added the number in the Fig. 1d. Thank you.*

7. Coefficient of variation means that SD/Mean (Standard deviation/Mean). The CCV (compartment coefficient of variation) was defined as the natural logarithm of the number of B compartments divided by the number of A compartments in AB variable compartment bins. In PAGE 6, LINE 44-50, I think the CCV does not indicate a coefficient of variation. I suggest using other abbreviations to describe the algorithm.

*【Response】Thanks for the reviewer’s suggestions. We have replaced it with ‘Coefficient of Compartment Variation’.*

8. Because the CCV is defined as B compartment divided by A compartment, it is positively correlated with E1. If CCV means B compartment divided by A compartment, the relation is negative. So I do not know why the author considers this result is "interesting"? In PAGE 6, in line 52.

*【Response】We think this discovery is interesting because: (1) AB variable compartments are mainly distributed in the intersection area of A/B compartments, which is closely related to the I region. Additionally, we proved that AB variable compartments are also significantly enriched in the I region; (2) the correlation between CCV and E1 suggested that the smaller the absolute value of E1 is, the easier the chromatin compartment is to be variable.*

*We are sorry that the word “interesting” may exaggerate the results. Following your suggestion, we have changed the text as “We further plotted AB variable compartments with CCVs throughout the genome, and observed that AB variable compartments were mainly distributed in regions of A/B compartment intersection, and the CCVs appeared to correlate with E1 values—the first eigenvalues in the eigenvector decomposition of compartments (Additional file 1: Fig. S3c).”*

9. Why two lines of insulation score are shown in figure 3a? What does each of them mean?

*【Response】We are sorry that we didn’t described these two lines in the figure legend. Referring to previous methods (Crane et al., 2015), the two lines represent insulation score (purple) and insulation delta score (yellow) respectively. We have added this description in figure legend. Thank you.*

*References*

*Crane, E., Bian, Q., McCord, R.P., Lajoie, B.R., Wheeler, B.S., Ralston, E.J., Uzawa, S., Dekker, J., and Meyer, B.J. (2015). Condensin-driven remodelling of X chromosome topology during dosage compensation. Nature 523, 240-244.*

10. I do not know how to identify the compartment border in LINE 56-58.

*【Response】We define ‘compartment border’ as the edge bin of the A/B compartments. We have added a description in the Methods.*

11. In the comparative 3D genome of TAD boundaries sections, how to convert the genome coordinate in page 21?

*【Response】The genome coordinate conversion method was described in the section ‘Genome coordinate conversion’ of Methods. Briefly, we convert the genome coordinates of TAD boundaries between the ZH13 reference genome and the query genomes using CrossMap with the option ‘CrossMap.py region’.*

*“Genome coordinate conversion*

*To convert the genome coordinates of TAD boundaries between the ZH13 reference genome and the query genomes, we first generated custom UCSC chain files between the reference genome and query genomes using the UCSC liftOver tool (http://genome.ucsc.edu). Thereafter, we performed genome coordinate conversion using CrossMap (v0.2.9) [76] with the option ‘CrossMap.py region’.”*

12. In page 22, what clustering algorithm was used for boundary clustering?

*【Response】Because we didn’t find any applicable algorithm for cluster the similarity results of 729 comparison files, we used our private code to merge the results. In detail, we compare the files one by one, and connect the relationship of TAD boundaries one by one. For example, if boundary A was connected with boundary B in file1, and boundary B was connected with boundary C in file2, we connected Boundary A, B and C after clustering, etc. The codes of this part have been uploaded to GitHub.*

13. "Although some SVs may not change the properties of A/B compartments, they affected the observed E1 values, such as the two large translocations on chromosome 11 and chromosome 13 in SoyW02 (Fig. 5b)", in PAGE 10. This conclusion needs statistic results to illustrate.

*【Response】Thank you for the good advice. We have further added the eigenvalues of A/B compartments to Fig. 5b and performed statistics of the E1 values. We have added the statistic analysis results in the main text.*

*“In addition, although some SVs may not change the properties of A/B compartments, they affected the observed E1 values, taking the two large translocations on chromosome 11 and chromosome 13 in SoyW02 as examples (Fig. 5b). For the first translocation pair (beginning ~20 Mb of SoyW02 chromosome 11 vs. end ~20Mb of ZH13 chromosome 13), the average E1 value from SoyW02 for this region was approximately 25% higher than that of ZH13 (0.65 ± 0.40 vs. 0.51 ± 0.18; mean ± s.d; P=1.295ⅹ10-5, paired t.test). For the second translocation pair (end ~5 Mb of SoyW02 chromosome 13 vs. end ~5Mb of ZH13 chromosome 11), although the E1 values from these two regions did not show significantly difference (0.81 ± 0.26 vs. 0.84 ± 0.19; mean ± s.d; P=0.068, paired t.test), but the E1 values from the last three windows of SoyW02 were approximately doubled compared to that of ZH13 (Fig. 5b).”*

14. Which 3D genomic structures are conserved and which are specific between wild soybeans, landraces, *and improved cultivars?*

*【Response】Appreciate you so much for this constructive comment. Following you suggestion, we have added the analysis ‘3D genome dynamics during domestication and improvement’.*

*“3D genome dynamics during domestication and improvement*

*It has been reported that cultivated soybean was domesticated from wild soybean (G. soja Sieb. & Zucc.) in China 5,000 years ago [39]. Apart from studies of genetic variations in the genome, little is known about the variability in higher-order chromatin structure during domestication and improvement in soybeans. To investigate this, we performed comparative analysis of the 3D genome variations in the wild soybeans (G. soja), landraces and improved cultivars.*

*Using the pan-3D genome data, we observed a core set of over 1,800 TAD boundary clusters shared by all three soybean groups. In addition, 457, 521, and 1,091 specific TAD boundary clusters were identified for individual G. soja, landrace and improved cultivar groups (Additional file 1: Fig. S10a). Combining with pairwise population differentiation level (FST) across different soybean groups, we found the selection of TAD boundaries was higher than that of Non-boundaries during the soybean domestication; whereas, no significant difference during improvement (Additional file 1: Fig. S10b). Further investigation suggested that the genes around selected TAD boundaries had higher expression levels than those of unselected TAD boundaries (Additional file 1: Fig. S10c).*

*We found the expression variations of some domesticated genes were closely related to the 3D genomic variations. For example, SoyZH13_14G139200 was located in a domesticated selection sweep region (Additional file 1: Fig. S10e). Structural variations led to the loss of the TAD boundary in two wild soybeans SoyW02, SoyW03 and one landrace SoyL02 (Additional file 1: Fig. S10d and e). As a result, we observed that the SoyZH13_14G139200 showed significant higher expression level in these 3 accessions than the others. These data suggested that 3D genome structure variation may also play important roles in soybean domestication and improvement.”*

15. The relationship between 3D genome variation and gene expression is an interesting scientific question. I would like to have a more in-depth study that does not stop at the level of statistical analysis of data and replication of known results but is preferably related to specific biological questions.

*【Response】Thank you for the suggestions. We have added more specific cases in the section of ‘3D genome variation and gene expression’.*

*“We further detected specific cases by correlating the 3D genome variation to the gene expression variation. For example, SoyZH13_09G153300 did not show obvious genetic variations in SoyC10 and SoyC11, but SoyC10 and SoyC11 showed significant lower gene expression than the other accessions (Additional file 1: Fig. S9a). Pan-3D genome showed that SoyC10 and SoyC11 lost one TAD boundary, which was ~60 kb away from this gene. As a validation, the Hi-C contact map showed that these losses led to a significant reduction in interaction, indicating it might cause the expression change in these two accessions. Another example was SoyZH13_19G056700, of which loss of one TAD boundary may also resulted in a lower expression of this gene (Additional file 1: Fig. S9b).”*

16. The authors construct 3D genome maps for wild soybean, landraces and improved cultivars, but did not perform comparison between these groups. I suggest they perform this analysis to explore the changes of 3D genome organization during domestication.

*【Response】Appreciate you so much for this constructive comment. Same as Question 14, following you suggestion, we have added the analysis ‘3D genome dynamics during domestication and improvement’.*

Reviewer #2

In a previous work, the authors generated a pan-genome resource of soybean. In this work, the authors presented pan-3D genome of soybean using Hi-C sequencing. Since the diverse accessions had high-quality genome assemblies, the relationship between 3D genomic variation and structural variations were well analyzed. Besides, in-depth analyses enabled the finding of the links between 3D variation and gene expressions. Generally, this is a very nice work and provides an important resource for further investigations.

*【Response】Thank you so much. We sincerely appreciate your encouragement.*

Below are several minor suggestions for the manuscript:

1. The Abstract of the manuscript should be rewritten, because many important results (e.g., the links between 3D variation and gene expressions) were not included.

*【Response】Thank you so much for pointing out this problem. We have rewritten the Abstract as you suggested.*

2. Page 5 Line 41, "some chromosomes in some accessions". The fuzzy descriptions should be revised.

*【Response】Thank you so much for pointing out this problem. We have replaced the sentence with ‘Nevertheless, we surprisingly found that the repeat percentage of the A compartment on several chromosomes was significantly higher than the average level, such as chromosome 1 of SoyC06’.*

3. Page 8, Line 2, "Fisher's exact test, p < 2.2 × 10-16". 2.2 × 10-16 is a threshold? It should be noted in the main text. Moreover, p should be italic.

*【Response】Thank you so much for this suggestion. We use R to perform statistics, P < 2.2 × 10-16 is the statistical result of P value in R. We are so sorry that we didn't describe it clearly in the last version, and realize that the list of the numbers in the main text may confuse the readers. We have deleted these in the main text, but keep them in the figures and give detailed description in the figure legend. Thank you again.*

4. More gene-level examples, especially the well-known genes in soybean, could be added in the section of "3D genome variation and gene expression" and "Effect and contribution of SVs to 3D genome variations".

*【Response】Appreciate you so much for this constructive comment. We have added these data of the results ‘3D genome variation and gene expression’ and ‘3D genome dynamics during domestication and improvement’.*

*“We further detected specific cases by correlating the 3D genome variation to the gene expression variation. For example, SoyZH13_09G153300 did not show obvious genetic variations in SoyC10 and SoyC11, but SoyC10 and SoyC11 showed significant lower gene expression than the other accessions (Additional file 1: Fig. S9a). Pan-3D genome showed that SoyC10 and SoyC11 lost one TAD boundary, which was ~60 kb away from this gene. As a validation, the Hi-C contact map showed that these losses led to a significant reduction in interaction, indicating it might cause the expression change in these two accessions. Another example was SoyZH13_19G056700, of which loss of one TAD boundary may also resulted in a lower expression of this gene (Additional file 1: Fig. S9b).”*

*“3D genome dynamics during domestication and improvement*

*It has been reported that cultivated soybean was domesticated from wild soybean (G. soja Sieb. & Zucc.) in China 5,000 years ago [39]. Apart from studies of genetic variations in the genome, little is known about the variability in higher-order chromatin structure during domestication and improvement in soybeans. To investigate this, we performed comparative analysis of the 3D genome variations in the wild soybeans (G. soja), landraces and improved cultivars.*

*Using the pan-3D genome data, we observed a core set of over 1,800 TAD boundary clusters shared by all three soybean groups. In addition, 457, 521, and 1,091 specific TAD boundary clusters were identified for individual G. soja, landrace and improved cultivar groups (Additional file 1: Fig. S10a). Combining with pairwise population differentiation level (FST) across different soybean groups, we found the selection of TAD boundaries was higher than that of Non-boundaries during the soybean domestication; whereas, no significant difference during improvement (Additional file 1: Fig. S10b). Further investigation suggested that the genes around selected TAD boundaries had higher expression levels than those of unselected TAD boundaries (Additional file 1: Fig. S10c).*

*We found the expression variations of some domesticated genes were closely related to the 3D genomic variations. For example, SoyZH13_14G139200 was located in a domesticated selection sweep region (Additional file 1: Fig. S10e). Structural variations led to the loss of the TAD boundary in two wild soybeans SoyW02, SoyW03 and one landrace SoyL02 (Additional file 1: Fig. S10d and e). As a result, we observed that the SoyZH13_14G139200 showed significant higher expression level in these 3 accessions than the others. These data suggested that 3D genome structure variation may also play important roles in soybean domestication and improvement.”*

**Second round of review**

**Reviewer 1**

The authors have addressed all my concerns, and the manuscript is largely improved now. This manuscript describes the first pan-3D genome study relative to crop domestication, which should serve as a good reference for other plants. Congratulations to all the authors.

**Reviewer 2**

The authors have well addressed all of my concerns and suggestions.
